# Identification of genes associated with cortical malformation using a transposon-mediated somatic mutagenesis screen in mice

**DOI:** 10.1038/s41467-018-04880-8

**Published:** 2018-06-27

**Authors:** I-Ling Lu, Chien Chen, Chien-Yi Tung, Hsin-Hung Chen, Jia-Ping Pan, Chia-Hsiang Chang, Jia-Shing Cheng, Yi-An Chen, Chun-Hung Wang, Chia-Wei Huang, Yi-Ning Kang, Hsin-Yun Chang, Lei-Li Li, Kai-Ping Chang, Yang-Hsin Shih, Chi-Hung Lin, Shang-Yeong Kwan, Jin-Wu Tsai

**Affiliations:** 1 Institute of Brain Science, National Yang-Ming University, Taipei, 112 Taiwan; 2 Department of Neurology, Neurological Institute, Taipei Veterans General Hospital, Taipei, 112 Taiwan; 3 National Yang-Ming University School of Medicine, Taipei, 112 Taiwan; 4 VYM Genome Research Center of National Yang-Ming University, Taipei, 112 Taiwan; 5 Institute of Microbiology and Immunology, National Yang-Ming University, Taipei, 112 Taiwan; 6 Department of Neurosurgery, Neurological Institute, Taipei Veterans General Hospital, Taipei, 112 Taiwan; 7 Taiwan International Graduate Program (TIGP) in Molecular Medicine, National Yang-Ming University and Academia Sinica, Taipei, 112 Taiwan; 8 Department of Pediatrics, Taipei Veterans General Hospital, Taipei, 112 Taiwan; 9 Institute of Biophotonics, National Yang-Ming University, Taipei, 112 Taiwan; 10 Brain Research Center, National Yang-Ming University, Taipei, 112 Taiwan; 11 Biophotonics and Molecular Imaging Research Center, National Yang-Ming University, Taipei, 112 Taiwan

## Abstract

Mutations in genes involved in the production, migration, or differentiation of cortical neurons often lead to malformations of cortical development (MCDs). However, many genetic mutations involved in MCD pathogenesis remain unidentified. Here we developed a genetic screening paradigm based on transposon-mediated somatic mutagenesis by in utero electroporation and the inability of mutant neuronal precursors to migrate to the cortex and identified 33 candidate MCD genes. Consistent with the screen, several genes have already been implicated in neural development and disorders. Functional disruption of the candidate genes by RNAi or CRISPR/Cas9 causes altered neuronal distributions that resemble human cortical dysplasia. To verify potential clinical relevance of these candidate genes, we analyzed somatic mutations in brain tissue from patients with focal cortical dysplasia and found that mutations are enriched in these candidate genes. These results demonstrate that this approach is able to identify potential mouse genes involved in cortical development and MCD pathogenesis.

## Abstract (web summary)

Cortical malformations have a variety of causes. Here the authors use transposon mutagenesis to insert mutations into neural stem cells in the developing mouse cortex to screen for new candidate genes for cortical malformation, and validate some targets in human brain tissue.

## Introduction

Development of the mammalian cerebral cortex is a complex dynamic process that can be broken down into a number of partially overlapping stages during gestation. The neuroepithelial cells (NEPs) first form the pseudostratified neural tube and subsequently transform into radial glia cells (RGCs) as cortical neurogenesis starts^1,2^. Cortical neurons generated directly or indirectly from RGCs in the ventricular zone (VZ) then migrate along radial fibers to form the highly organized cortical layers^3–5^. The timing and dynamics of these cellular processes require precise genetic regulations; any perturbations may lead to cortical malformations^6–8^. In human, malformations of cortical development (MCDs) often result in pediatric neurological dysfunctions presented by epilepsy, intellectual disability, developmental delay, and even autism^9^.

To date, the genetic causes of a number of MCDs have been identified, including microcephaly (e.g., *MCPH1*, *ASPM*, *CPAP*, *CDK5RAP2*, and *STIL*), lissencephaly (e.g., *LIS1*, *DCX*, *ARX*, and *TUBA1A*), double cortex (e.g., *DCX*), periventricular nodular heterotopia (e.g., *ARFGEF2*), and tuberous sclerosis (e.g., *TSC1* and *TSC2*)^10–12^. Recently, deep whole-exome sequencing (WES) has uncovered somatic mutations of genes involved in the mTOR pathway to cause focal cortical dysplasia (FCD)^13–16^, a form of MCDs characterized by localized cortical malformation^17^. However, many other genes potentially involved in cortical development and MCD pathogenesis remain unidentified^15^.

To identify new genes potentially involved in cortical development and the pathogenesis of MCDs, we took advantage of forward genetic screening by somatic mutagenesis during brain development. Previously, transposons have been utilized for mutagenesis attributed to their ability to randomly insert into the genome and cause mutations. Particularly, *piggyBac* (PB), a transposon originally found in the cabbage looper moth *Trichoplusia ni*, shows high mobility when introduced into mammalian cells^18^. In addition, the insertion sites in the genome are relatively easy to identify—compared to radiation and chemical mutagenesis. Thus PB is an effective way of forward genetic screens in a range of model organisms^19–21^.

Here we developed an in vivo genetic screen paradigm that utilizes in utero electroporation of PB transposon into mouse embryos to induce insertional mutations in RGCs. Using this method, we identified dozens of candidate genes potentially involved in cortical development. Combining with bioinformatics analysis, RNA interference (RNAi) and CRISPR/Cas9 technologies, we were able to verify their potential roles in the development of the cerebral cortex. To explore the clinical relevance of these candidate genes, we analyzed the somatic mutations present in brain tissue from human FCD patients and found that somatic mutations coincide with many of these candidate genes. These findings demonstrate that our method is able to identify new genes that are involved in cortical development and associated with neurodevelopmental disorders.

## Results

### An animal model of MCD by transposon insertional mutagenesis

**Fig. 1:**
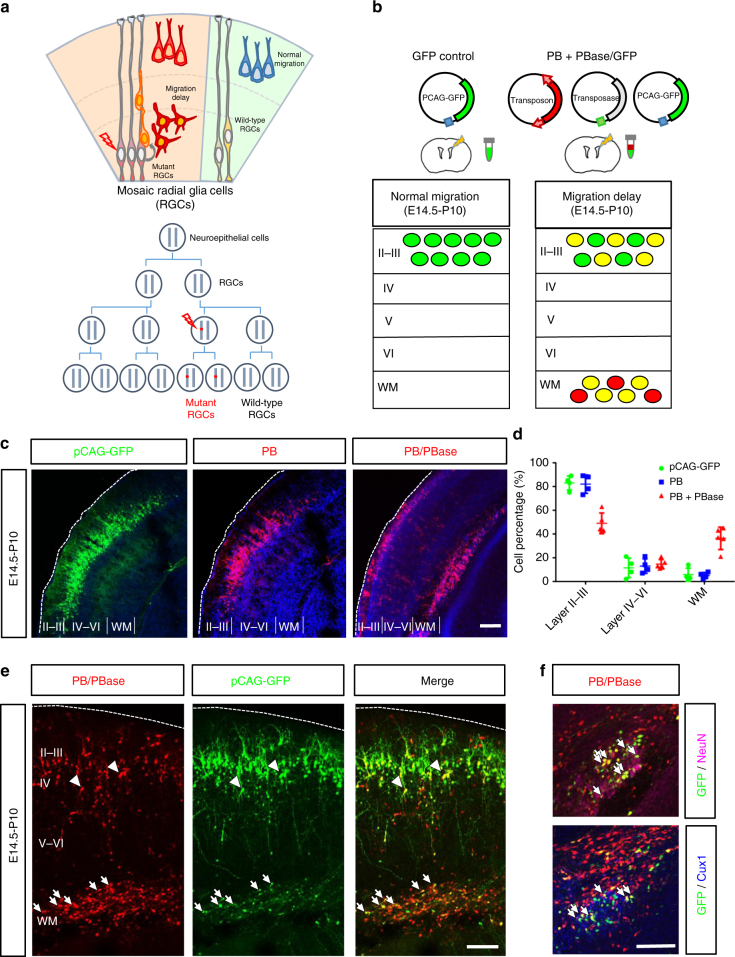
Altered neuronal distribution during cortical development by *piggyBac* transposon mutagenesis. **a** Top: a schematic diagram of effects of somatic mutations in cortical development. While normal GRCs produce neurons that migrate normally to the cortex, RGCs that carry detrimental mutations may cause migration delay of their progeny. Bottom: early somatic mutation events lead to a large number of mutant cells carrying the same mutation through clonal expansion. **b** A schematic diagram of the PB-induced neuronal migration delay in the developing mouse cortex. The brains were electroporated with GFP (green) alone or with PB and PBase (red) at E14.5 and the distribution of cells was assessed at P10. **c** In mouse brains electroporated with pCAG-GFP (green, left panel) or PB (red, center panel) at E14.5, the labeled cells were primarily found in layer 2/3 of the cerebral cortex at P10. In contrast, an additional ectopic layer of cells was found beneath the cortex in brains electroporated with PB and PBase (right panel), this combination allowing the insertion of PB into the genome. Bar = 200 μm. **d** Cell distributions in different brain regions of the cerebral cortex. Control pCAG-GFP: *n* = 4 animals; PB: *n* = 4 animals; PB + PBase: *n* = 6 animals. *: *p* < 0.05, Student’s *t* test. Error bars = s.d. **e** Arrest of GFP+ cells by PB insertional mutagenesis. Brains were electroporated with pCAG-GFP, PB, and PBase at E14.5 and examined at P10. GFP and RFP double positive cells were observed not only in the cortex (arrowheads) but also ectopically in the WM (arrows). Bar = 100 μm. **f** Neuronal identify of cells arrested in the subcortical regions. Many GFP (green) and RFP (red) double positive cells in the brain slices expressed the neuronal marker NeuN (magenta, arrows, upper panel) and layer 2–4 marker Cux1 (blue, arrows, lower panel) at P10. Brain slices were co-stained with DAPI (blue). WM, white matter. Bar = 100 μm

To identify potential genes involved in cortical development, we established a genetic screening paradigm by combining transposon mutagenesis and in utero electroporation (Fig. 1). The transposable element PB with its corresponding transposase were delivered into neural stem cells (i.e., RGCs) in the developing neocortex by in utero electroporation at embryonic day 14.5 (E14.5), at which time cortical neurogenesis was most active^22^. Under the activity of transposase, the transposon could potentially cause insertional mutations in the genomes of neural stem cells and, subsequently, their progenies (Fig. 1a). When the insertion occurs within a gene, it likely results in disrupted or hypomorphic alleles due to the large size (~11 kb) of the inserted PB. As the normal progeny cells differentiate and migrate to the cortex, cells carrying mutations that cause defects in neuronal development and/or migration would stay in the VZ and SVZ after birth. This altered cell distribution allowed us to isolate defective cells and identify potential genes important for this process (Fig. 1b).

To induce insertional mutagenesis and observe mutant cells, we used the PB-3R-puro construct^18,23,24^, which encodes a PB transposable element carrying a red fluorescence protein (RFP) for visualization of electroporated cells by fluorescent microscopy. When the brains were electroporated with PB alone or a control pCAG-GFP construct expressing green fluorescence protein (GFP) at E14.5, the fluorescently labeled cells were primarily found within layers 2/3 and 4 of cortex on postnatal day 10 (P10) (Fig. 1c, d). The labeled cells extended multiple dendrites toward the pial surface and a single axon that projected toward other brain regions, in agreement with previous studies^25^. When PB and its corresponding transposase PBase were co-transfected, an additional population of RFP+ cells was found in the white matter (WM) ectopically. Interestingly, most of the ectopic RFP+ cells appeared as multipolar and resembled immature neurons. We therefore hypothesized that at least some of the cells localized in the deeper layers were likely to result from defects in neuronal development caused by transposon insertion.

When PB is inserted into the genome of RGCs, all their progeny will be labeled with RFP, including the early-generated neurons in the cortex and the late-generated glial cells in the WM^26^. To test whether the RFP+ cells found in the WM were ectopic neurons arrested by PB-induced migration delay but not WM-destined glial cells, we co-electroporated pCAG-GFP, which only labels early-generated neurons, into embryonic mouse brains (Fig. 1e). We found that, while most of the GFP+ cells were found in layers 2/3 and 4 of the neocortex at P10, some GFP and RFP double positive cells were found in the WM of the neocortex (Fig. 1e) compared to cells with PB or PBase alone, which were found almost only in layers 2/3 and 4 (Supplementary Fig. 1a, b). In order to test the identity of the arrested cells, brain slices were stained with the neuronal marker NeuN and marker for layer 2–4 neurons Cux1^27^. We found that many of the GFP+/RFP+ cells were NeuN+ and Cux1+ (Fig. 1f, Supplementary Fig. 1c, d), indicating a neuronal lineage. These above results indicate that some PB insertions into the genome of neural progenitors are sufficient to arrest postmitotic neurons ectopically in the WM.

### Identification of candidate MCD genes by splinkerette PCR

**Fig. 2:**
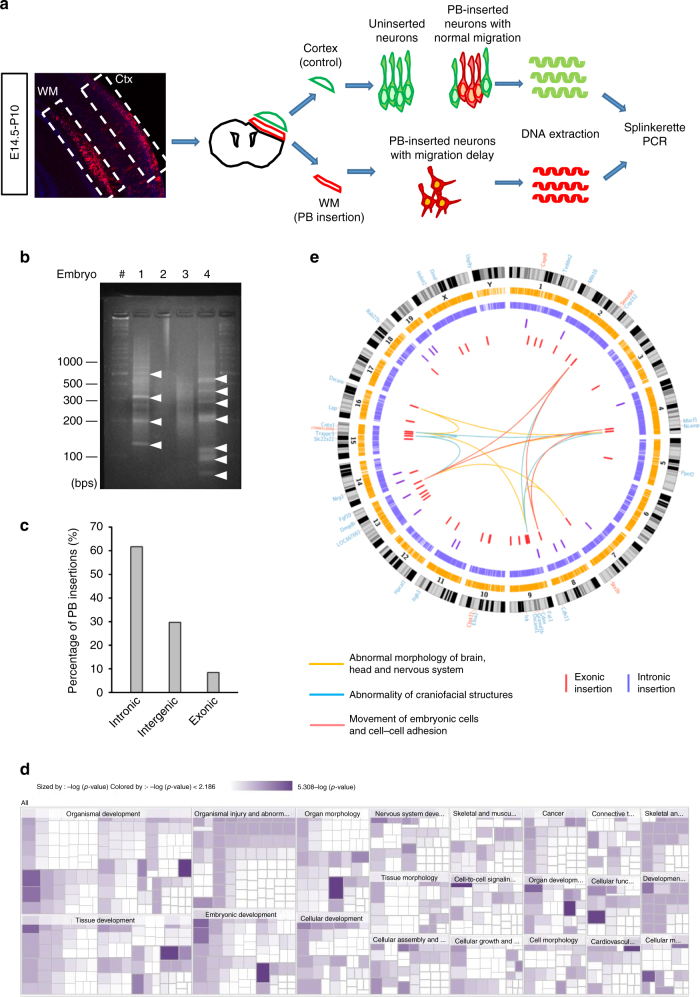
Identification of candidate genes involved in cortical development via PB-based insertional mutagenesis. **a** The procedure used for the genetic screening. Cortical and ectopic layers containing cells electroporated with PB/PBase (red) at E14.5 were isolated surgically at P10. Genomic DNA (gDNA) was then extracted for splinkerette PCR to identify the insertion sites. **b** The products of the splinkerette PCR from four different mouse brains separated by agarose gel electrophoresis. Each prominent band (arrowheads) was excised for DNA sequencing. **c** Distribution of PB insertions within 50 kb of known genes in the mouse genome. Percentage of the PB insertions located in exons, introns, 5′ intergenic regions and 3′ intergenic regions are shown. **d** IPA was used for the functional categorization of the candidate genes. The area of each rectangle represents the *p*-value of a particular gene relevant to a particular functional pathway. The results show that these genes are mainly involved in development and nervous system functions. **e** A Circos diagram illustrating the positions of the identified candidate genes within the mouse genome and the type of insertion (intron and exon). Genes involved in three pathways were also linked together; these were genes involved in abnormal morphology of the brain, head and nervous system (yellow strings), genes involved in abnormalities of the craniofacial area, rhombencephalon and telencephalon (blue strings), and genes involved in cell movement of embryonic cells as well as cell–cell adhesion (red strings)

To analyze the PB insertional mutations that caused the defects in neuronal distribution, we first surgically isolated the brain regions containing the ectopic RFP+ cells and extracted the genomic DNA from this region (Fig. 2a). Tissue containing RFP+ cells that had migrated to the cortex was also collected as a control. The DNA from dissected tissues was then subjected to splinkerette-PCR, which allows amplification of the targeted genomic sequences in conjunction with the long terminal repeats (LTR) on both sides of the transposon^28,29^. We collected 754 major bands from PCR products containing insertion site fragments of genomic DNA from 855 neonates at P10 for Sanger sequencing (Fig. 2b). The major PCR bands presumably came from progeny cells that were derived from stem cells with early insertion events and had undergone clonal expansion. A number of 504 of the products yielded readable sequences, which were then compared with the mouse genome database using NCBI BLAST and the Ensemble genome browser (www.ensemble.org). We found that 11.5% (58/504) of the insertion sites lied within 50 kb of known genes. Among them, 6.8% (4/58) and 65.6% (38/58) of the insertion sites were in exons and introns, respectively, while 27.6% (16/58) were intergenic (Fig. 2c). In total, 42 genes were found to contain PB insertional mutations within the genome of the ectopic neurons (Supplementary Table 1). Among them, two genes (*Cdon* and *Cops8*) were found to be mutagenized at different sites in different animals.

To exclude insertions that also appeared in neurons found in the cortex, we also analyzed the PB insertion sites in the genomes of RFP+ cells from the cortical region using the same method. Again, at least 31 genes were found to contain PB insertional mutations within the genome of the normal neurons (Supplementary Table 2, Supplementary Data 1). The insertion sites of nine genes overlapped with those found in ectopic neurons within the WM (Supplementary Table 2). We therefore excluded these genes from our candidate gene pool, thus yielding 33 candidate genes that are potentially important for neuronal proliferation, differentiation and/or migration during cortical development (Table 1). We further confirm the expression of these 33 candidate genes in the cerebral cortex based on previous literature^30–34^, Allen Brain atlas (http://www.brain-map.org/), and immunofluorescence staining (Supplementary Fig. 2). The available data showed that at least 23 of them are expressed in the developing cortex (Supplementary Table 1).

**Table 1:** Genes identified from PB-based in vivo genetic screen of cortical development

Chr	Gene name	NCBI reference Sequence	Description	Insertion position	Pathway	Human disease
**1**	*Cops8*	NM_133805.3	COP9 signalosome subunit 8	Exon	Deneddylation of the cullin subunits of SCF type E3 ligase complexes	
**1**	*Teddm2*	NM_178243.3	Transmembrane epididymal family	Intron		
**2**	*Sema6d*	NM_172537.4	Semaphorin 6d	Exon	Collapse of growth corn, maintenance and remodeling of neuronal connections	
**2**	*Mllt10*	NM_010804.4	Myeloid/lymphoid or mixed-luneage leukemia, translocated 10	Intron	Chromosomal rearrangements resulting in various leukemias	
**4**	*Macf1*	NM_001199136.1	Microtubule-actin cross-linking factor 1	Intron	Rearrangement of epithelial cells, neurite outgrowth and guidance	
**4**	*Ncmap*	NM_145555.2	Noncompact myelin associated protein	Intron		
**5**	*Ppef2*	NM_011148.3	Protein phosphatase EF hand calcium-binding domain 2	Intron		
**7**	*Stx1b*	NM_024414.2	Syntaxin 1B	Exon	Turnover of synaptic vesicles	Generalized epilepsy with febrile seizures plus type 9
**8**	*Cdh13*	NC_000074.6	Cadherin-13	Intron	Cell–cell adhesion	
**9**	*Cdon*	NM_021339.2	Cell adhesion molecule-related/down regulated by oncogenes	Intron	Enlargement of esophagus, cell-cell adhesion, formation of skull	Holoprosencephaly type 11
**9**	*Gramd1b*	NM_172768.1	GRAM domain containing 1B	Intron		
**9**	*Dscaml1*	NM_001081270.1	Down syndrome cell adhesion molecular like 1	Intron	Cell–cell adhesion	
**9**	*Fat3*	NC_000075.6	Protocadherin Fat 3	Intron	Interactions between neurites	
**9**	*Ick*	NM_001163780.1	Intestinal cell kinase	Intron	Flagellar formation, ciliary length control	Endocrine-cerebro-osteodysplasia
**10**	*Efna2*	NM_007909.3	Ephrin-A2	Intron	Migration, repulsion and adhesion during neuronal, vascular and epithelial development	
**10**	*Chst11*	NM_021439.2	Carbohydrate sulfotransferase 11	Exon	Catalyzes the transfer of sulfate to position 4 of the *N*-acetylgalactosamine (GalNAc) residue of chondroitin	
**11**	*Itgb3*	NM_016780.2	Integrin beta 3	Intron	Outgrowth of organoid, cell-cell adhesion, developmental process of synapse, retraction of cytoplasmic matrix	
**12**	*Hpcal1*	NM_016677.4	Hippocalcin-like 1	Intron	Calcium-dependent regulation of rhodopsin phosphorylation	
**13**	*Cep152*	NM_00181091.1	Centrosomal protein of 152	Intron	Centrosome duplication	Seckel syndrome 5/autosomal recessive microcephaly type 9
**13**	*Dmgdh*	NM_028772.3	Dimethylglysine dehydrogenase	Intron	Betaine degradation and in amine and polyamine degradation	Dimethylglycine dehydrogenase deficiency
**13**	*Fgf10*	NM_008002.4	Fibroblast growth factor 10	Intron	Development of cervical loop proliferation of transitional epithelium, formation of skull	Microcephaly
**13**	*LOC667693*	NC_000079.6	Uncharacterized protein LOC667693	Intron		
**14**	*Nrg3*	AF010130.1	Neuregulin 3	Intron	Direct ligand for the ERBB4 tyrosine kinase receptor, stimulated tyrosine phosphorylation and activation of the receptor	
**15**	*Cntn1*	NM_001159647.1	Contactin-1	Intron	Abnormal morphology of Golgi interneurons, developmental process of synapse, formation of inhibitory synapse	Compton-North congenital myopathy
**15**	*1700001L05Rik*	NC_000081.6	RIKEN cDNA 1700001L05	Intron		
**15**	*Trappc9*	NM_180662.2	Trafficking protein particle complex subunit 9	Intron	An activator of NF-kappa-B through increased phosphorylation of the IKK complex, neuronal cells differentiation, vesicular transport from endoplasmic reticulum to Golgi	Autosomal recessive mental retardation type 13
**15**	*Slc22a22*	NM_172378.2	Solute carrier family 22	Intron	Sodium-independent organic anion transmembrane transporter activity	
**16**	*Dscam*	NM_031174.4	Down syndrome cell adhesion molecule	Intron	Neuronal self-avoidance, repulsion between specific neuronal processes	Down syndrome, congenital heart disease
**16**	*Lpp*	NM_178665.5	LIM domain containing preferred translocation partner in lipoma	Intron	Cell adhesion in maintaining cell shape and motility	
**18**	*Rab27b*	NM_030554.4	Member RAS oncogene family Rab27b	Intron	Docking of multivesicular bodies, targeting uroplakins to urothelial apical membranes	
**X**	*Hs6st2*	NC_000086.7	Heparin sulfate 6-*O*-sulfotransferase	Intron	Modification of heparan sulfate proteoglycan	Hypoplasia of lacrimal gland
**X**	*Dmd*	EF067597.1	Domesticus isolate MWN1291 dystrophin	Intron	Anchors the extracellular matrix to the cytoskeleton via F-actin. Ligand for dystroglycan	Duchenne muscular dystrophy with mental retardation
**Y**	*Usp9y*	NM_148943.2	Ubiquitin specific peptidase 9	Intron	Ubiquitin-dependent protein catabolic process	Y-linked spermatogenic failure type 1

### Bioinformatics analysis of identified potential FCD genes

To obtain insights into the biological functions of these 33 candidate genes and how they are related to each other, Ingenuity Pathway Analysis (IPA, http://www.ingenuity.com) based on biomedical literature was used to carry out a bioinformatics analysis (Supplementary Data 2). We found that the highest associated biological functions of these candidate genes were: (1) organismal development, (2) tissue development, (3) organismal injury and abnormalities, (4) embryonic development, (5) cellular development, (6) nervous system development, (7) tissue morphology, and (8) cellular assembly and organization (Fig. 2d; Supplementary Table 3; Supplementary Data 2). This analysis suggested that our candidate gene list was indeed highly associated with development. As far as human diseases are concerned, many of these genes are associated with developmental disorders, in particular craniofacial abnormalities, such as holoprosencephaly type 11 (*CDON*)^35^, microcephaly (*CEP152*, *FGF10*)^34,36^, Seckel syndrome 5 and primary microcephaly type 9 (*CEP152*)^37^, Duchenne muscular dystrophy with mental retardation and the absence of ERG b-wave (*DMD*)^38^, autosomal recessive mental retardation type 13 (*TRAPPC9*)^33^, generalized epilepsy with febrile seizures plus type 9 (*STX1B*)^39^ and Compton-North congenital myopathy (*CNTN1*)^40^.

To illustrate the distribution of the insertion sites in the mouse genome, we labeled each mutation site on a circular layout and the results show that the insertions do not display any preference in terms of chromosome location (Fig. 2e). We further grouped these genes in terms of their involvement in abnormal morphology of the brain, head and nervous system (*CDON*, *CNTN1*, *DSCAM*, *EFNA2*, and *FGF10*; yellow strings); in abnormality of craniofacial region, rhombencephalon and telencephalon (*CEP152*, *FGF10*, *TRAPP9*, and *CNTN1*; blue strings); and cell movement of embryonic cells and cell–cell adhesion (*FGF10*, *SEMA6D*, *CDH13* and *MACF1*; red strings). These analyses demonstrated that our PB-mediated forward genetic screen was able to identify genes potentially involved in the development of nervous system and in neurological disorders.

### Validation of candidate genes affecting neuron distribution

To validate involvement of the candidate genes in cortical development and to further investigate their roles in this process, we used in utero electroporation to express short-hairpin RNAs (shRNA) and knockdown these candidate genes in neural progenitor cells during cortical development^41–43^. We used previously validated shRNA constructs, available from the National RNAi Core Facility in Academia Sinica, Taiwan, to target 25 of the 33 identified genes (Supplementary Table 4). We also confirmed the knockdown efficiency in cultured cortical neurons infected with lentiviruses carrying shRNA sequences (Supplementary Fig. 3). In addition, we tested the effects of depleting 7 of the 9 genes that were excluded in our screen, as an additional control (Supplementary Table 4).

**Fig. 3:**
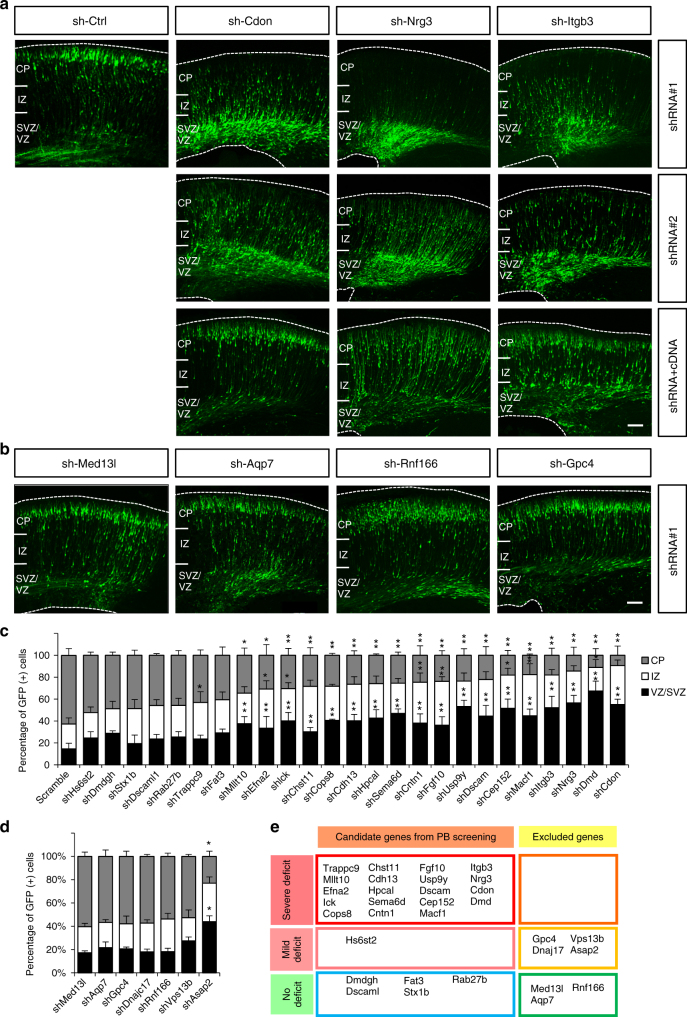
Phenotype in neuronal distribution after knockdown of candidate genes during brain development. **a** Distribution of cells in brains electroporated with shRNAs against candidate genes at E14.5 and imaged 4 days later at E18.5. Two shRNAs against each genes were used for each genes. Brains electroporated with shRNAs targeting *Cdon*, *Nrg3*, and *Itgb3* all altered neuron distribution with the accumulation of GFP+ cells in the VZ/SVZ compared to the control brain. Overexpression of these genes rescue the phenotype caused by shRNA (bottom row). **b** Knockdown of the excluded genes produced more limited effects. Bar = 100 μm. **c** Distributions of cells in the brains electroporated with shRNA targeting 25 genes from the screening. *n* = 3 animals for each group. *: *p* < 0.05, **: *p* < 0.01, Student’s *t* test. Error bars = s.d. **d** Distributions of cells in the brains electroporated with shRNA targeting seven of the excluded genes from the screening. *n* = 3 animals for each group. *: *p* < 0.05, Student’s *t* test. Error bars = s.d. **e** Summary of cell distribution phenotypes by shRNAs targeting the candidate and excluded genes

Brains from E14.5 mouse embryos were electroporated with two specific shRNAs targeting each candidate gene or a scramble control-shRNA, together with pCAG-GFP. The brains were harvested 4 days after electroporation at E18.5 and cell distribution was analyzed by confocal microscopy. (Fig. 3, Supplementary Figs. 4, 5). In control brains, the majority of neural precursor cells had migrated from the VZ to the CP by day 4. In contrast, brains electroporated with 19 of the 25 shRNA constructs (72 %) targeting these candidate genes (e.g., *Cdon*, *Nrg3*, and *Itgb3*) exhibited severe abnormal distribution of neurons, with significantly increased numbers of cells in the VZ and IZ (Fig. 3a; Supplementary Fig. 4a). The distributions of the knockdown cells in the shRNA-transfected brains all were significantly different from the situation in the control brain (*p* < 0.05, Student’s *t* test; Fig. 3c; Supplementary Fig. 4b). In contrast, all of the 7 shRNA constructs in the excluded gene list exhibited only mild or no effect in cell distributions compare to the control (Fig. 3b, d; Supplementary Fig. 5). Therefore, these results indicated that depletion of genes from the screening paradigm has a higher probability to cause defects in cortical development (Fig. 3e).

To minimize the off-target effect of shRNA, we overexpressed shRNA-resistant versions of the candidate genes in the most severe groups, including *Cdon*, *Nrg3*, *Itgb3*, *Cep152*, *Dscam*, and *Cntn1*. We found that expression of each of these genes was able to partially rescue the phenotypes in migration delay caused by shRNA, further confirming their potential roles in brain development (Fig. 3a; Supplementary Fig. 4c).

### Somatic mutations in candidate genes from FCD patients

**Fig. 4:**
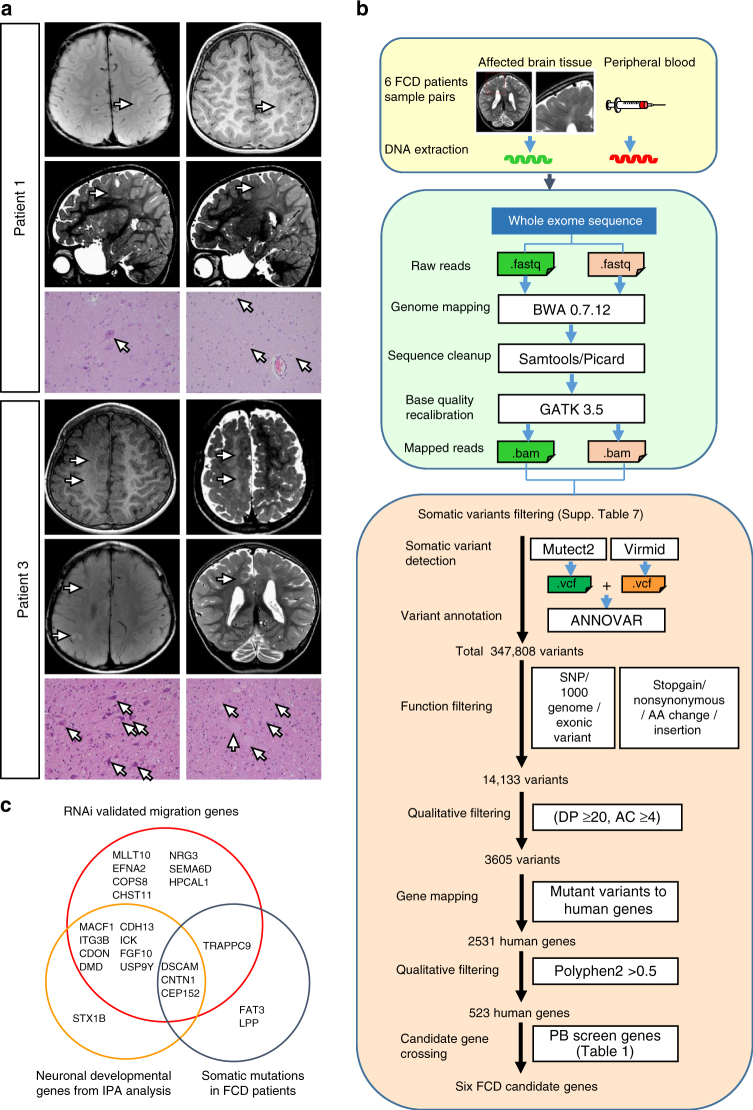
WES analysis of somatic mutations in blood/tissue pairs from FCD II patients. **a** The pre-surgery brain T1 and T2 MRI images of two FCD II subjects showing the lesion sites (arrows). H&E staining of pathological samples showed dysmorphic and cytomegalic neurons (balloon cells) (arrows). **b** Flow chart of the WES pipeline and data analysis by the Virmid and MuTect algorisms. DP, Read depth, AC, alternative allele counts. **c** Overlap of gene mutations found in human FCD patients, genes involved in neural development from IPA analysis and genes validated using RNAi

In recent years, somatic mutations in neural cells have been found in the lesion tissue from patients with FCD^13–16,44^. We therefore sought to test whether somatic mutations in the 33 candidate genes using our screening paradigm could also be found in the brain tissue of FCD patients. Six FCD type II patients from Taipei Veterans General Hospital (VGH), who were diagnosed with early onset epilepsy (4 d/o–5 y/o) and had no family history of this disease, were enrolled in the study (Table 2). The brain tissue samples obtained after therapeutic surgery all showed the characteristic pathological features of FCD type IIb, namely dysmorphic neurons or balloon cells (Fig. 4a)^6^. The genomic DNA from freshly frozen brains and the blood tissues from each patient was then sequenced using deep WES (Fig. 4b; Supplementary Table 5).

**Table 2:** Clinical data of FCD of patients

Patient #	Sex	Age	Age of onset	Seizure type	Age of surgery	Lesion	Pathology
1	M	5y6m/o	4 d/o	Asymmetric tonic	3y3m/o	Left rolandic	Type IIb
2	M	11 y/o	2 y/o	Focal motor	3 y/o	Left high parietal	Type IIb
3	M	7 y/o	7 m/o	Epilepsia partialis continua	1st 3y6m/o 2nd 4y3m/o	Right F-C-P	Type IIb
4	M	20 y/o	5 m/o	Automotor	17 y/o	Right P	Type IIb
5	F	10 y/o	8 m/o	Generalized tonic	2y7m/o	Right P	Type IIb
6	F	16 y/o	5 y/o	Focal tonic, dyscognitive	12 y/o	Right F, insula	Type IIb

*F-C-P* fronto-centro-parietal, *P* parietal, *F* frontal

**Fig. 5:**
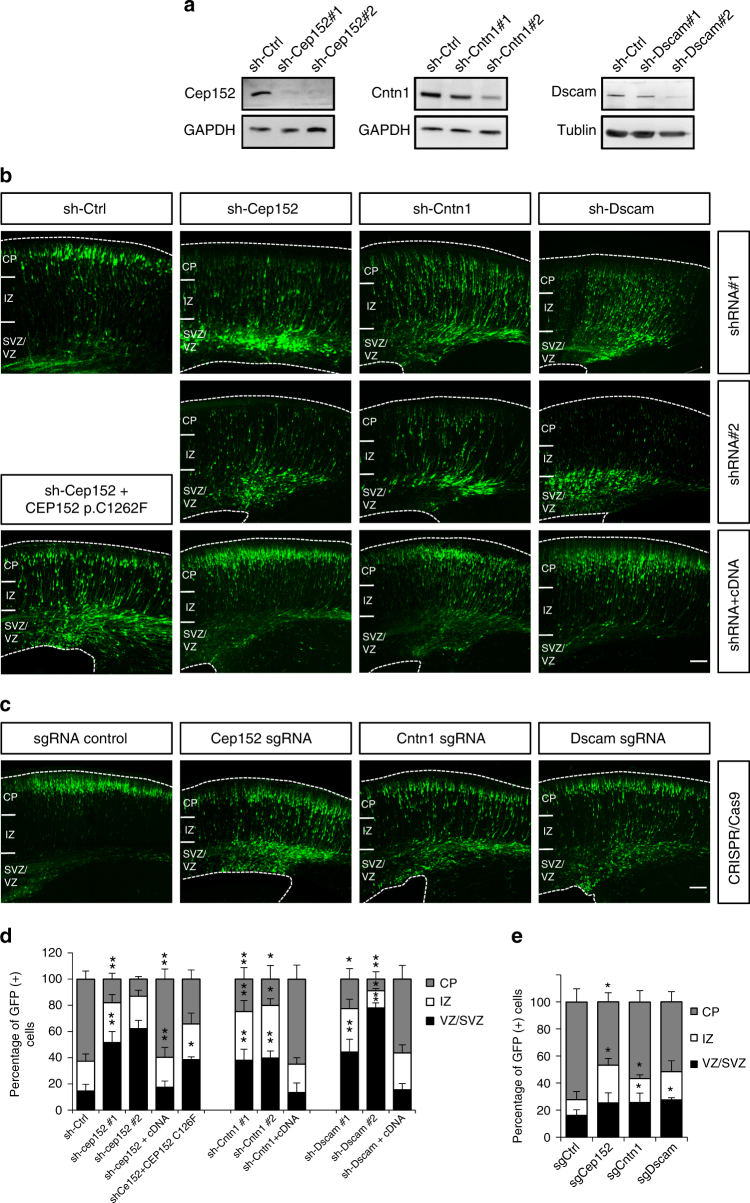
Neuronal distribution after knockdown and disruption of candidate genes. **a** Knockdown efficiency of shRNAs targeting candidate genes by western blot. **b** Distribution of cells in brains electroporated with shRNAs against candidate genes at E14.5 and imaged 4 days later at E18.5. Two shRNAs against each genes were used for each genes. Brains electroporated with shRNAs targeting *Cep152*, *Cntn1*, and *Dscam* all altered neuron distribution with the accumulation of GFP+ cells in the VZ/SVZ compared to the control brain. **c** Overexpression of these genes rescue the phenotype caused by shRNA in the same condition. **d** Distributions of cells in brains electroporated with shRNAs, and rescue constructs. *n* = 3 animals for each group. *: *p* < 0.05, **: *p* < 0.01, Student’s *t* test. **e** Disruption of these three genes with CRISPR/Cas9 technology using electroporation of sgRNA also showed ectopic GFP+ cells in the SVZ/VZ and IZ. *n* = 3 animals for each group. *: *p* < 0.05, Student’s *t* test. Bars = 100 μm

To detect rare somatic mutations in the affected brain tissue, we utilized two somatic variants detecting algorithms, Virmid (http://sourceforge.net/projects/virmid/) and MuTect (http://www.broadinstitute.org/cancer/cga/mutect), which have been commonly used for the detection of low allelic frequency mutations in heterogeneous tissues, including those from FCD patients^45–47^ (Fig. 4b). The identified potential somatic variants (Supplementary Data 3) then went through quantitative functional filters to remove low quality reads, reported SNPs, and non-exonic variants (Fig. 4b, Supplementary Table 6, Supplementary Data 4). Among these variants, we found that 6 of them (*CEP152*, *CNTN1*, *DSCAM*, *FAT3*, *LPP*, and *TRAPPC9*) lied within the 33-candicate gene set (Supplementary Fig. 6; Supplementary Data 4). Interestingly, 3 genes (*CEP152*, *CNTN1*, and *DSCAM*) overlapped with neurodevelopmental genes from IPA analysis (Fig. 4c). Knocking down each of them by RNAi all showed migration delay in the developing mouse brain (Fig. 5a, b, d). Moreover, to determine whether the mutations identified in FCD patients would cause protein loss of function, we examined whether expression of mutant proteins can rescue the migration defects in knockdown neurons. RNAi-resistant CEP152 or its p.Cys1262Phe mutation, which was predicted as damaging by SIFT, was electroporated to Cep152-knockdown neurons. We found that while overexpression of *CEP152* cDNA restored the migration delay in Cep152-knockdown neurons, CEP152 p.Cys1262Phe was not able rescue this defect (Fig. 5b, d).

**Fig. 6:**
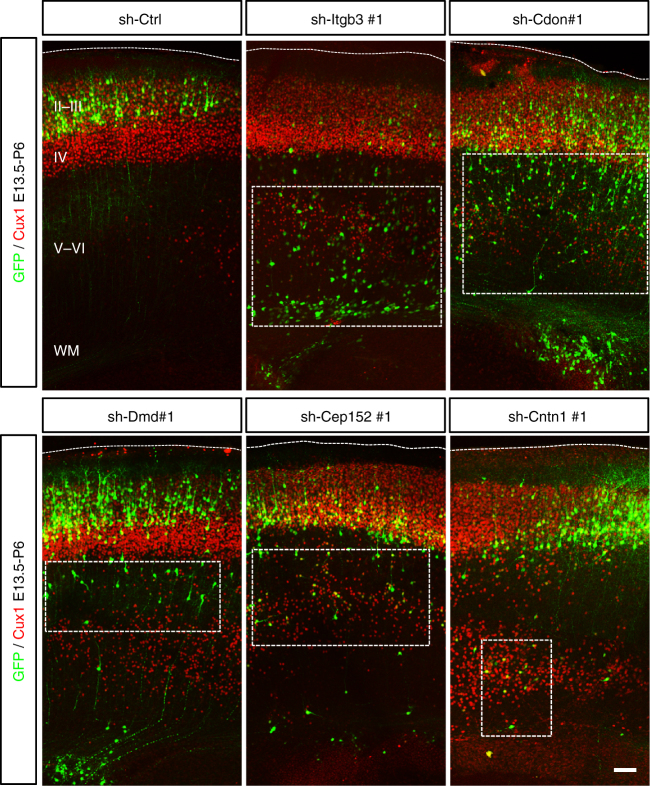
Ectopic neurons of P6 brains electroporated with shRNAs targeting MCD candidate genes. Brains were electroporated with shRNAs at E14.5 and fixed at P6. Marker of layer 2–4 cells, Cux1 (red), were used for cell identities. In control brains, neurons have reached layers 2/3 and 4 of the cortex by P6. However, brains electroporated with shRNAs targeting *Itgb3*, *Cdon*, *Dmd*, *Cep152*, and *Cntn1* along with GFP (green) exhibited different numbers of ectopic neurons underneath layer 4 (boxes)

To determine whether direct disruption of these genes during cortical development could result in migration dysfunction, we employed CRSIPR/Cas9 system^48,49^ to induce small indels in the genomes of RG cells (Fig. 5c). We designed small guide RNAs (sgRNAs) targeting the PB-insertion sites of these three genes (*Cep152*, *Cntn1*, and *Dscam*; Supplementary Table 7). We first found that treatment with Cas9 and these sgRNAs reduced mRNA and protein levels of targeted genes in cultured cortical neurons (Supplementary Fig. 3b; Supplementary Table 7). Remarkably, brains electroporated with vectors carrying the Cas9 and sgRNAs of each of these 3 genes all showed accumulation of neurons in the IZ and SVZ (Fig. 5c, e). Furthermore, when the brains electroporated with shRNA against these candidate genes were examined at P6, many neurons were found beneath layer 2/3, resembling ectopic neuron layers of many MCD pathology (Fig. 6). Altogether, our results indicate that the screen paradigm combining with bioinformatics and human patient mutations was able to identify genes important for cortical development and malformations.

## Discussion

**Fig. 7:**
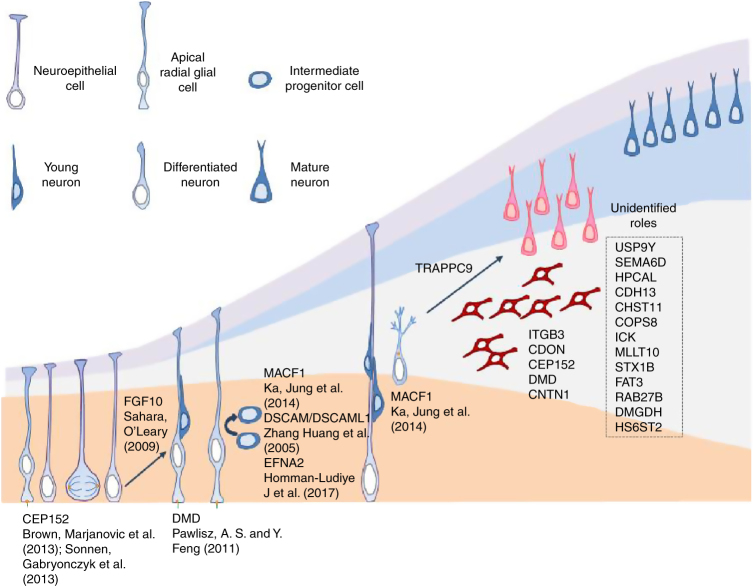
Potential cellular roles of the candidate MCD genes involved in cortical development RGCs divide to self-renew and produce neural precursors in the VZ/SVZ. The progeny cells then migrate along radial fibers to the CP and differentiate into neurons. Known (with references) roles for the candidate MCD genes are indicated. The roles of potential new genes in cortical development on right remain to be determined

In this study, we developed a mouse model based on transposon-mediated mutagenesis and in utero electroporation to screen for candidate genes involved in cortical development (Fig. 1), and identified 33 candidate genes (Table 1). Bioinformatics analysis demonstrated that these candidate genes are highly associated with neuronal development and various neuronal disorders (Fig. 2). Dysfunction of many of these genes by RNAi caused defects in neuron distribution during cortex development (Fig. 3). We further used WES with paired blood and brain samples from FCD type II patients (Table 2) to identify somatic mutations and found 6 mutated genes overlapped with our candidate gene set (Fig. 4). Disruption of these genes using CRISP/Cas9 technology leads to arrest of cells in the cortex, further validating the essential roles of these genes in cortical development (Fig. 5). This genetic screening paradigm therefore serves as a useful tool for the identification of new genes that are involved in cortical development and these mutations are likely to be related to neurological disorders such as FCD (Fig. 7).

Forward genetics has been widely utilized in model systems using lower organisms including yeast, worm, fruit fly, and zebra fish. These organisms have relatively short life cycles and maintaining a large number of mutant organisms is not that costly. In terms of mammalian models, previous efforts have involved the use chemical mutagens, such as *N*-ethyl-*N*-nitrosourea (ENU), and established a number of libraries of mutant mice. Later, genetic screening after transposon-based or virus-based mutagenesis in mice has proven to be powerful for the identification of candidate cancer genes^50^. In some cases, cells electroporated with transposable elements were injected into animals^51^. Alternatively, transgenic mice carrying transposable elements have been crossed with mice carrying the corresponding transposase in order to induce tumorigenesis^52^. However, this powerful approach has not been applied to studies in cortical development.

In the current study, we used in utero electroporation to introduce PB and PBase into neural stem cells to generate somatic mutations in the developing cortex. Screening was performed based on the inability of these cells to relocate correctly within the cortex in vivo. The advantage is that each mouse carries a large number of random insertional mutations in the brain. By isolating cells arrested in the WM of neonates, one can isolate cells defective in proliferation, migration, and/or differentiation during cortical development. The insertion sites can be easily identified using splinkerette-PCR; therefore it is possible to obtain candidate mutations starting from electroporation within a few weeks, much faster than generating mutant mice.

Although this method could not generate stable mutant mice, it serves as a fast and cost-effective initial screen. The function(s) of these candidate genes can then be validated using RNAi, CRISPR/Cas9, and ultimately transgenic mice. Notably, knocking down any of the 19 candidate genes (out of 25 tested) from our list was sufficient to cause migration delay. Gene disruption using CRISPR/Cas9 system also showed a similar phenotype. Therefore, we believe that each gene detected by this method has a high probability of playing important roles in neuronal development.

Using this method, we identified genes not only involved in cortical development, but also relevant to human MCDs. Indeed, we found genes implicated in holoprosencephaly type 11 (*CDON*)^35^, microcephaly (*CEP152*, *FGF10*)^34,36^, Duchenne muscular dystrophy with mental retardation and the absence of ERG b-wave (*DMD*)^38^, autosomal recessive mental retardation type 13 (*TRAPPC9*)^33^, generalized epilepsy with febrile seizures plus type 9 (*STX1B*)^39^ and Compton-North congenital myopathy (*CNTN1*)^40^. We propose that mutations in other candidate genes may worth further investigation in human MCD patients.

Furthermore, this method generated mosaic mutations in the developing brain, which has been proposed as an important mechanism for FCD^13–15^. These mice exhibited ectopic dysmorphic neurons in the WM, which also resembles the hallmarks of FCD II pathology^53^. To date, many mutations related to FCD II lesion have been found to involve molecules associated with the mTOR pathway, including *MTOR*, *AKT3*, *PIK3R2*, and *PIK3CA3*. However, in our FCD II patients we identified new mutations in six genes that appeared on the MCD candidate gene list identified by our screening paradigm. More importantly, the mutation enrichment in the candidate gene list is significantly higher than that in randomly selected 33 genes among 20,000+ genes in the human genome (*p* = 0.001). As a comparison, 7 mutations were found in 51 members of the mTOR pathway (*p* = 0.134), including *MTOR* and *AKT* (Supplementary Data 4).

In the analysis of somatic mutations in FCD brain tissues, hundreds to thousands of potential variants are normally found, as in our patients (Fig. 4c). It is therefore very difficult to narrow down disease causing genes that contribute to the pathogenesis of the lesion. Previously, genes in the MTOR pathway have served as a powerful panel to identify potential mutations in causing FCD and hemimegalencephaly^13–15^. Here we propose that using the gene set identified from our screen can also be useful to find potential mutations contributing to FCD and other brain developmental disorders in human.

Using this screen approach, we uncovered a number of new potential molecules involved in different stages of cortical development (Fig. 7). For example, some of these candidates are involved in cell cycle progression and stem cell differentiation, such as *Cep152*^54,55^ and *Fgf10*^34^. Disruption of their function may result in abnormal proliferation of neural progenitor cells. Indeed, using staining of cell cycle markers, we found a decrease cell cycle exit in brains electroporated with *Cep152* and *Fgf10* shRNAs, supporting their roles in cell cycle regulation (Supplementary Fig. 7). Some genes may be involved in differentiation of neurons, such as *Trappc9*^56^ and *Cntn1*^57;^ others involved in neuronal migration, such as *Dscam*, *Dscaml1*^58^, *Macf1*^59^, and *Efna2*^60^. In addition, some are also implicated in neurite/axonal growth, such as *Sema6d*^61^, *Macf1*^62^, *Cntn1*^63^, *Dscam*^58^, and *Dmd*^64^ (Table 1). Their involvement in a variety of neuronal functions is consistent with their expression in the brain (Supplementary Table 1, Supplementary Fig. 2). Defects in these processes will likely affect cortical development during our genetic screening. Indeed, RNAi for the many of these candidate genes were found to create defects of varying severity in the distribution of neurons within the cortex. Therefore, further investigations are needed to shed light on the cellular and molecular functions of these proteins during cortical development.

In summary, we have established a genetic screening paradigm that is able to effectively identified known and new genes involved in cortical development. Mutations in many of these genes are found in MCD and FCD type II patients and represent potential pathological causes of cortical malformation.

## Methods

### Constructs

The PB transposon system consisted a piggyBac transposon with the PB-tk-mRFP reporter and the Act-PBase helper plasmid of Act-PBase; these were provided by J-Y Lee^18,23,24^. The shRNA constructs, which are based on the pLKO_TRC001 vector, were obtained from the National RNAi Core Facility in Taiwan (Supplementary Table 4). The knockdown efficiency of these constructs was pre-validated and this has been reported previously by the National RNAi Core Facility, Taiwan. The CRISPR/Cas9 and sgRNA constructs were obtained from the National RNAi Core Facility in Taiwan. sgRNA sequences were designed by their online protocols (Supplementary Table 7). The human full-length cDNA for rescue experiments were purchased from the Mammalian Gene Collection (MGC) premier clone (TransOMIC technologies).

### Animal model and in utero electroporation

Timed pregnant C57BL/6 and ICR mice were used for the in utero electroporation at E14.5. The procedure for in utero electroporation has been described previously^25,43^ and followed the approval protocol together with the Rodent Survival Surgery Guidelines and Affidavit by the Institutional Animal Care and Use Committee at National Yang-Ming University (NYMU). Briefly, mice were anesthetized with 2% isoflurane. An incision was made through the skin and the abdominal muscle in order to expose the underlying viscera. Each embryo was randomly injected with 0.5 μl of a secretly coded solution containing the appropriate DNA into one side of the lateral ventricle of the brain. Electroporation involving five 50 ms pulses with an interval of 450 ms at a voltage of 50 V was then carried out. After electroporation, the uterine horns were placed back into the abdominal cavity carefully and the incision was closed by suture. The embryos were allowed to develop normally in the uterus and birth by the mother occurred naturally. The brains of the electroporated mice were harvested at E18.5, P6, or P10. Animals appearing healthy with normal activity and without obvious weight loss were all included in the analysis. However, brains with low electroporation efficiency (<10^4^ cell/brain) were excluded. The person who analyzed the brain images were blinded to the prior manipulations.

### Immunofluorescence staining

The neonates collected after electroporation were fixed by transcardial perfusion of 4% paraformaldehyde and the brains were sectioned by Vibratome (Leica). When necessary, the brain slices were subjected to heat-induced epitope retrieval; this involved placing the slices in sodium citrate buffer solution (pH 6.0) and heating them up to 95 °C or heating them in a microwave oven for 10 min. This was followed by blocking with 10% normal goat/donkey serum, 2% bovine serum albumin, 0.2% Triton X-100 in phosphate-buffered saline (PBS) for 1 h at room temperature. Primary antibodies were then added and the mixture incubated at 4 °C overnight. The concentration of primary antibodies: NeuN (1:500, Millipore, Cat.# MAB377), anti-Cux1 rabbit polyclonal antibody (1:500; Santa Cruz Biotechnology, sc-13024), anti-5-bromo-2′-deoxyuridine (BrdU) rat monoclonal antibody (1:250; Abcam, Cat.# ab6326), anti-Ki67 rabbit polyclonal antibody (1:500; Millipore, AB9260), anti-Dmd rabbit polyclonal antibody (1:500, Proteintech, Cat. #:12715-1-AP), anti-Rab27b rabbit polyclonal antibody (1:500, Proteintech, Cat. #:13412-1-AP), anti-Dmghd rabbit polyclonal antibody (1:500, Proteintech, Cat. #:24813-1-AP), anti-Hapcal rabbit polyclonal antibody (1:500, Proteintech, Cat. #:10989-1-AP), anti-Rnf166 rabbit polyclonal antibody (1:500, ABclonal, Cat. No.: A8276), anti-Cep152 rabbit polyclonal antibody (1:500, Proteintech, Cat. #: 21815-1-AP), anti-Ick rabbit polyclonal antibody (1:500, Proteintech, Cat. #:13611-1-AP), anti-Stx1b mouse monoclonal antibody (1:500, Proteintech, Cat. #: 66437^−^1-lg), anti-Vps13b rabbit polyclonal antibody (1:500, Proteintech, Cat. #: 24505-1-AP), anti-Med13l rabbit antibody (1:500, AVIVA SYSTEM BIOLOGY, Cat. #: OASG07310). After washing the brain slices with PBS, they were then incubated with Alexa Flour-546-conjugated secondary antibody (1:500, Invitrogen) for 2 h at room temperature.

### Splinkerette PCR and sequence analysis

The procedure for Splinkerette PCR has been described previously. Briefly the long- and short-strand adaptors were mixed at a final concentration of 25 µM (Long-stand adaptor, CGAAGAGTAACCGTTGCTAGGAGAGACCGTGGCTGAATGAGACTGGTGTCGACACTAGTGG; short-strand adaptor, GATCCCACTAGTGTCGACACCAGTCTCTAATTTTTTTTTTCAAAAAAA) and allowed to anneal. Next 2 µg of genomic DNA was digested with 40U Sau3A1 in a total volume of 30 µl at 37 °C overnight (12–16 h). A mixture of 1 µl of annealed splinkerette adaptors, 4 µl of 10× DNA-ligation buffer, 1 µl of T4-DNA-ligase (20U), and 4.5U of Sau3A1 digested genomic DNA was then incubated overnight at 4 °C to allow ligation.

A QIAquick Gel Extraction Kit (Qiagen) was then used to purify the ligated products and remove excess adaptor and salts. Twice nested PCR reactions were then performed on each sample to allow splinkerette amplifications at the 5′ and 3′ ends. The results of the splinkerette PCR reaction (15–20 µl) were visualized by gel electrophoresis on a 4% agarose gel. The purified excised bands were subjected to PCR sequencing and the results were analyzed by NCBI BLAST searches of the mouse genome databases.

### RNA and protein knockdown assay

E14.5 mouse brains were harvested and dissociated with HBSS medium and placed in papain (20 unit/ml) with HBSS at 37 °C for 30 min. Cell were then centrifuged at 1000 *×* *g* for 5 min, resuspended with Neuronal basal medium (Life Technologies), B27 (Life Technologies), Glumatin max (Gibico), penicillin and streptomycin, supplemented with DNase and albumin-ovomucoid inhibitor. The culture dishes were coated with 1% (w/v) poly-d-lysine overnight and 5% Metrigel in artificial cerebrospinal fluid (ACSF) medium. Cells were seeded with 1.25 × 10^6^ cells and infected the virus expressing shRNA or sgRNA after DIV 5 day. Protein or RNA samples were then collected for western blot or q-PCR experiments. The antibodies used for western blot were as followed: anti-Cep152 rabbit polyclonal antibody (1:500, Proteintech, Cat. #: 21815-1-AP), anti-Cntn1 rabbit polyclonal antibody (1:500, Proteintech, Cat. #: 13842-1-AP), anti-Dscam rabbit polyclonal antibody (1:1000, Abcam, Cat. #: ab85362), anti-Cdon rabbit polyclonal antibody (1:500, GeneTex, Cat. #: GTX105422), anti-Dmd rabbit polyclonal antibody (1:500, Proteintech, Cat. #: 12715-1-AP), anti-Nrg3 goat polyclonal antibody (1:500, GeneTex, Cat. #: GTX89047), anti-Stx1b mouse monoclonal antibody (1:500, Proteintech, Cat. #: 66437-1-lg), anti-Dnaj17 rabbit polyclonal antibody (1:500, EnoGene, Cat. No: E11-11764C), anti-Rnf166 rabbit polyclonal antibody (1:500, ABclonal, Cat. No: A8276), anti-Gpc4 rabbit polyclonal antibody (1:500, Proteintech, Cat. #: 13048-1-AP).

The cDNA synthesis of RNA samples was performed by using iScript cDNA synthesis kit (Bio-Rad Laboratories). We used the StepOnePlus^TM^ Real-Time PCR System (Applied Biosystem) by following the qPCR protocol of OmicsGreen qPCR kit. The primers were designed as follows: M18S (normalization control) Fw TTC GAA CGT CTG CCC TAT CAA, M18S Rv ATG GTA GGC ACG GCG ACT A, Cep152 Fw GCA GGA AAC TGC ACG GAA GA, Cep152 Rv TTC TCA GCT CCC TCC TCT TTT C, Cntn1 Fw AGT CAA AAT TTC AGG CGT GTC C, Cntn1 Rv GCC GCA GGG ATT AGA AGG AA, Dscam Fw ACC CCT TCA GAA TCG GGG AT, Dscam Rv GTA TGA GGT CGC CTG GCC G.

### FCD subject identification

Patients diagnosed as FCD with epilepsy were enrolled in the study from Taipei VGH. All of the patients were fully notified and signed the informed consent approved by the Institutional Review Board (IRB) of Taipei VGH. FCD subjects were classified using diagnostic criteria based on the ILAE classification^6^ and admitted for a comprehensive pre-surgical evaluation before epilepsy surgery was carried out. The evaluation included recording detailed clinical history, video-EEG monitoring, neuroimaging (MRI), single photon emission computed tomography (SPECT), 18-F positron emission tomography (PET), functional MRI and DTI (when patients has a lesion that could not be disclosed by MRI); finally intracranial grid or strip implantation with further monitoring was used as necessary. The clinical characteristics of our six patients and their pathology are summarized in Table 2. Two of our six patients suffered from severe developmental delay (patients 1 and 3), while others had a borderline or normal mental status and IQ test results. All the surgical specimen of our patients had a pathological diagnosis of FCD type IIb as reported by a pathologist. These patients all were found to have dysmorphic neurons or balloon cells during the pathological examination. All patients selected were followed up for at least 2 years after surgery (4–8 years) and four of them remained seizure free (patients 1, 2, 4, and 5). The other two, while not seizure free, showed an improvement of more than 50% in both frequency and severity of seizures. Patient 3 underwent a second surgical procedure due to the presence of a large and diffuse lesion. After surgery two patients were able to stop taking antiepileptic medications (patients 2 and 5).

### Whole-exome sequencing

WES was performed using an Illumina HiSeq 2500 platform according to the manufacturer’s instructions. Genomic DNA was extracted from whole blood and from the affected brain tissue from the six FCD IIb patients. The quantified genomic DNA was then randomly fragmented to give molecules with a size peak between 150 and 200 bp and adapters were then ligated to both ends of these fragments. Exome-capture was performed using the SureSelectXT Human All Exon v.4 for Sample FCD1 and 3 (Agilent Technologies), and SeqCap_EZ_ExomeV3_Plus_UTR kit (NimbleGen Technologies) for Sample FCD 2, 4, 5, and 6, following the manufacturer’s specifications.

Alignment to the reference genomes (hg19 for human) was performed using Burrows–Wheeler Aligner (BWA). After Next-Generation Sequencing data pre-processing (local realignment, duplicate marking and base quality recalibration) using GATK3.5, we obtained a haploid mean coverage of 50–100×. We identified single nucleotide variants (SNVs) and small insertions/deletions (indels) in our samples using MuTect (http://www.broadinstitute.org/cancer/cga/mutect) and Virmid (http://sourceforge.net/projects/virmid/), respectively. We applied to the resulting variants the following additional filters: (i) the minimum read depth = 20 for both normal and tumor samples; (ii) the minimum number of alternative reads = 4. The identified 3605 variants were functionally annotated using ANNOVAR 4^65^. We excluded variants in non-coding regions, synonymous variants from further analysis. The mutation points were all validated by manually viewing in Integrative Genomics Viewer (Supplementary Fig. 6; http://software.broadinstitute.org/software/igv/). The whole-exome sequencing data have been deposited in the NCBI database under accession codes: BioProject ID: PRJNA471928; SRA database: SRP148319.

### Gene enrichment analysis

We performed gene enrichment analysis by hypergeometric test. The 3605 variants selected from somatic mutations detection were first annotated to corresponding genes by ANNOVAR^65^, yielding a total of 2531 genes. Because causal genes of FCD can be multiple or various among each patient, the patient vs. population frequency is not a sufficient criterium to exclude variants, neither is protein structure prediction. Therefore, we did not further narrow down the variant list by extra filter. We instead ask whether these 33 potential candidate genes are enriched in these 2531 potential causal gene of FCD using gene set enrichment analysis. The significance of gene enrichment was estimated by hypergeometric distribution function of R.

### Mutagenesis of CEP152 cDNA

Human CEP152 cDNA in the pCR-XL-TOPO vector was purchased from the MGC premier cDNA clone. The mutagenesis PCR protocol was conducted by following the instructions from the QuikChange II Site-Directed Mutagenesis Kit (Agilent Technologies, Cat. 200524). The primers used for CEP152 p.Cys1262Phe were: 5′-TCC TGC AAA ATC TGT TGG AGG AAA ATC AAA TAA TAT TTG C-3′(sense) and 5′-GCA AAT ATT ATT TGA TTT TCC TCC AAC AGA TTT TGC AGG A-3′(antisense).

### Cell-cycle exit analysis

After in utero electroporation, the ICR mother was injected intraperitoneally with BrdU (50 mg/g/body weight). After 24 h, embryos brain were fixed and processed as described above. Brain sections were incubated with 1 N HCL for 20 min at 56 °C for denature DNA, rinsed in 0.1 M sodium borate solution for 5 min to restore pH. The brain sections were blocked and incubated with anti-BrdU and Ki67 antibody for 48 h at 4 °C. Cell-cycle exit ratio was calculated as the fraction of BrdU^+^/Ki67^−^ among all electroporated GFP+ cells over the number of electroporated GFP+ labeled with BrdU (GFP^+^ and BrdU^+^)^66^.

### Statistical analysis

The datasets were analyzed using one-sided Student’s *t* test where appropriate. One-way ANOVA was used to analysis the differences among each group. Bonferroni post hoc test was used to analyze for significant differences in the ANOVA results. All data are expressed as mean ± s.d. Statistical significance was set at *p* *<* 0.05 (95% confidence level). For neuronal distribution studies, *n* ≥ 3 animals were analyzed for each group.

### Data availability

The datasets generated during and/or analyzed during the current study are available from the corresponding author on reasonable request. The NGS data were deposited into the Sequence Read Archive (SRA) database (accession number: SRP148319; BioProject ID: PRJNA471928) at the National Center for Biotechnology Information (NCBI).

## Electronic supplementary material

Supplementary Information

Description of Additional Supplementary Files

Supplementary Data 1

Supplementary Data 2

Supplementary Data 3

Supplementary Data 4
