# Supplementary material for: Identification of genes associated with cortical malformation using a transposon-mediated somatic mutagenesis screen in mice
Source: Nat Commun. 2018 Jun 27;9:2498. doi: 10.1038/s41467-018-04880-8 (PMC6021418; doi:10.1038/s41467-018-04880-8)
Supplement: Supplementary file 1 — Supplementary Information [file 41467_2018_4880_MOESM1_ESM.pdf]

Lu *et al.*

Identification of genes associated with cortical malformation using a transposon-mediated somatic mutagenesis screen in mice

Supplementary Figures and Tables

Lu *et al.*, Supplementary Figure 1

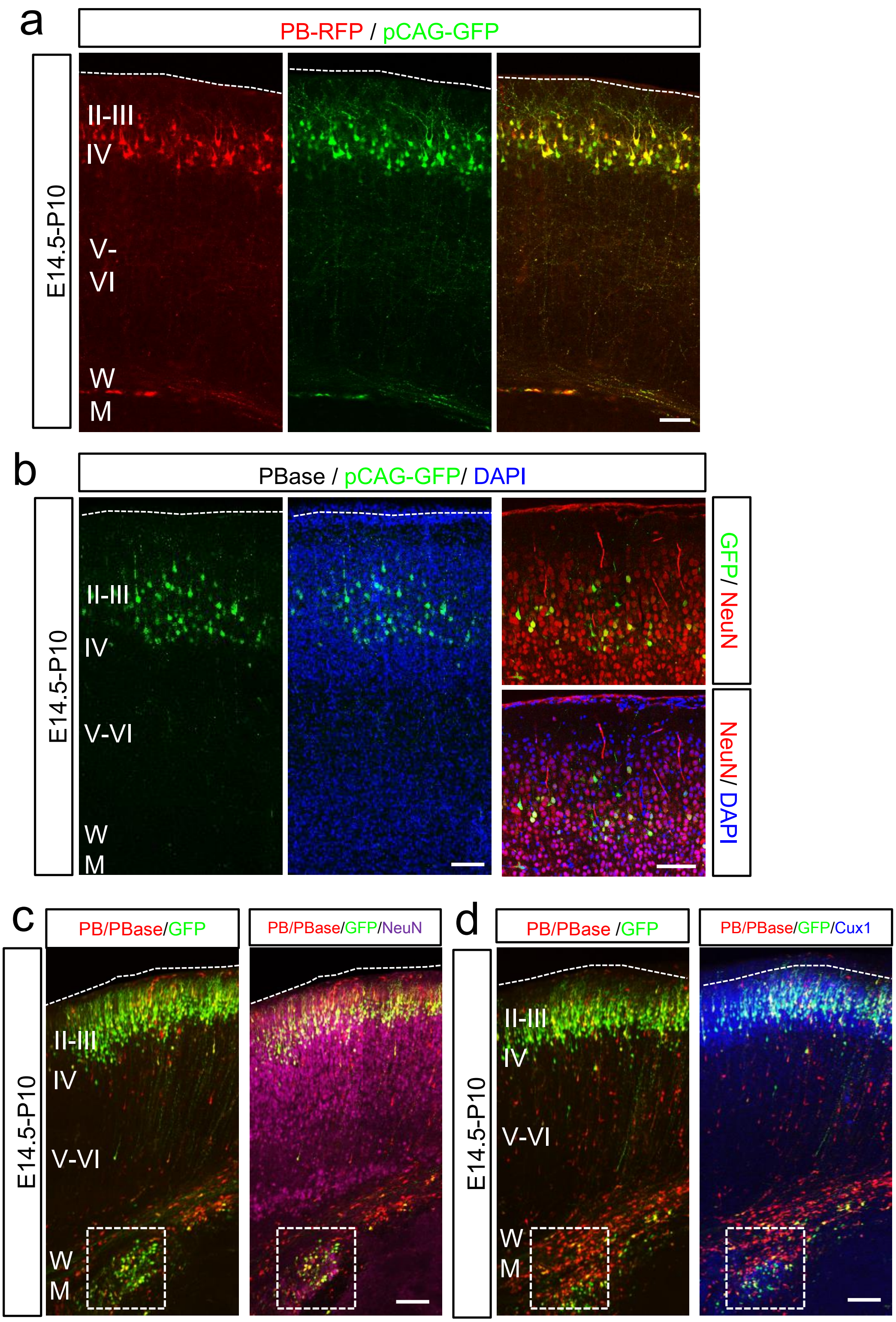

Supplementary Figure 1. Distribution of neurons in the mouse cortex at P10 after electroporation of pCAG-GFP with PB or PBase at E14.5 (a) Most pCAG-GFP (green) and PB (red) double positive cells are found in Layer 2/3 of the cortex and present with a typical neuronal morphology (*n* = 4 animals). Bar = 100 µm. (b) Cells electroporated with PBase are also found primarily in Layer 2/3 and are NeuN+ (right panels; *n* = 3 animals). Bars = 100 µm. (c) Neurons co-electroporated with PB/PBase (red) and GFP (green) stained with neuronal marker NeuN (magenta). Boxed area is shown in **Fig. 1c**. Bar = 100 µm. (d) Neurons co-electroporated with PB/PBase (red) and GFP (green) stained with layer 2-4 marker Cux1 (blue). Boxed areas are shown in **Fig. 1c**. Bar = 100 µm.

Lu *et al.*, Supplementary Figure 2

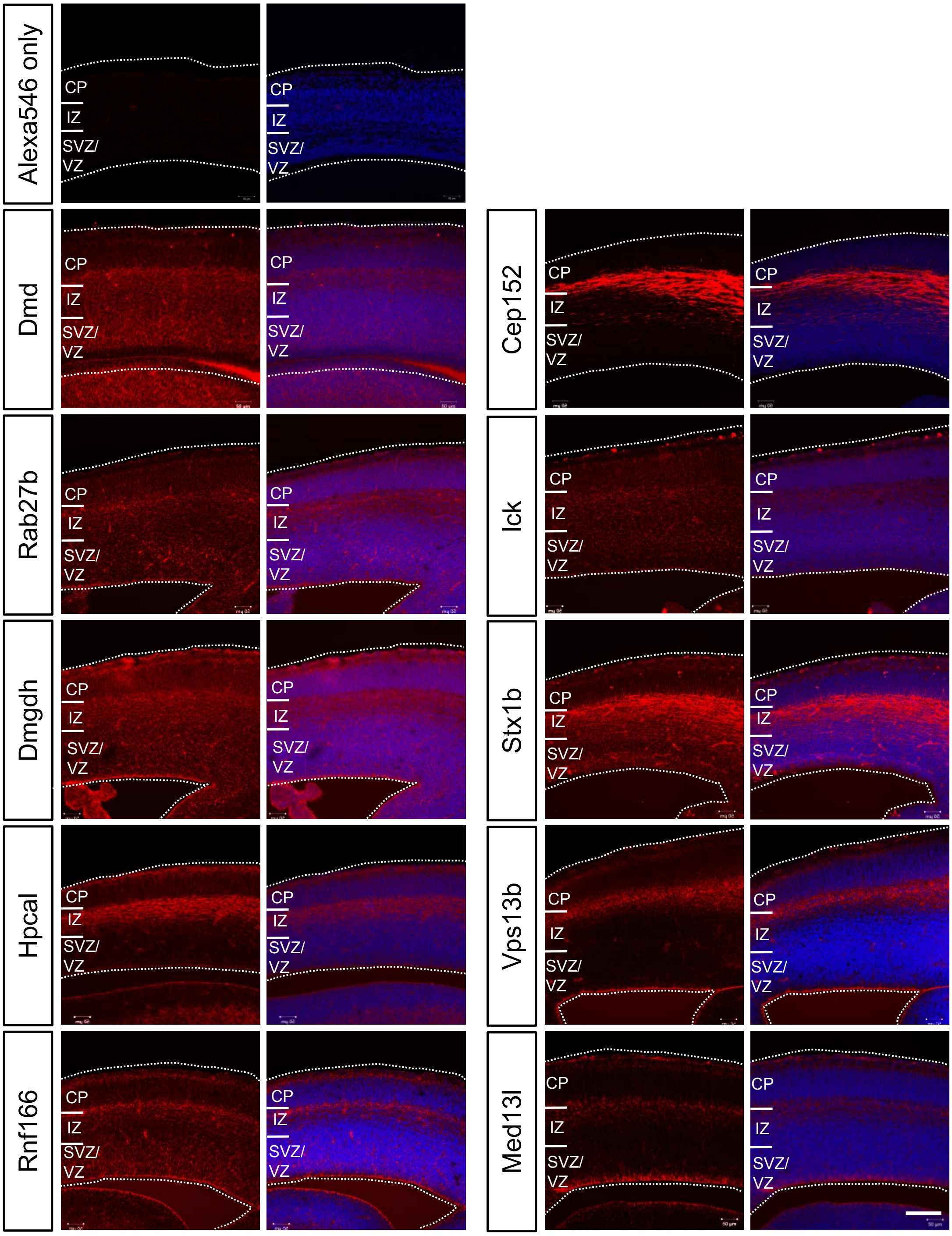

Supplementary Figure 2. Immunostaining of candidate genes in the developing E14.5 cortex. Fluorescence signals can be found with primary antibodies against most of the candidate genes. Some of the proteins were expressed throughout the entire developing cortex (e.g., Dmgdh and Ick). Some were predominantly in the IZ (e.g., Hpcal), implicating their roles in neuronal migration, while some were in both the IZ and VZ/SVZ (e.g., Rab27b, Rnf166, Stx1b, Vps13b and Med13l), suggesting their roles in both migration and neurogenesis. Interestingly, some also showed strong signals in the axonal area, in addition to IZ and VZ/SVZ (e.g., Dmd), implicating their additional functions in the axon. The staining is complementary to known protein expression in **Supplementary Table 1**. Bar = 100 μm.

Lu *et al.*, Supplementary Figure 3

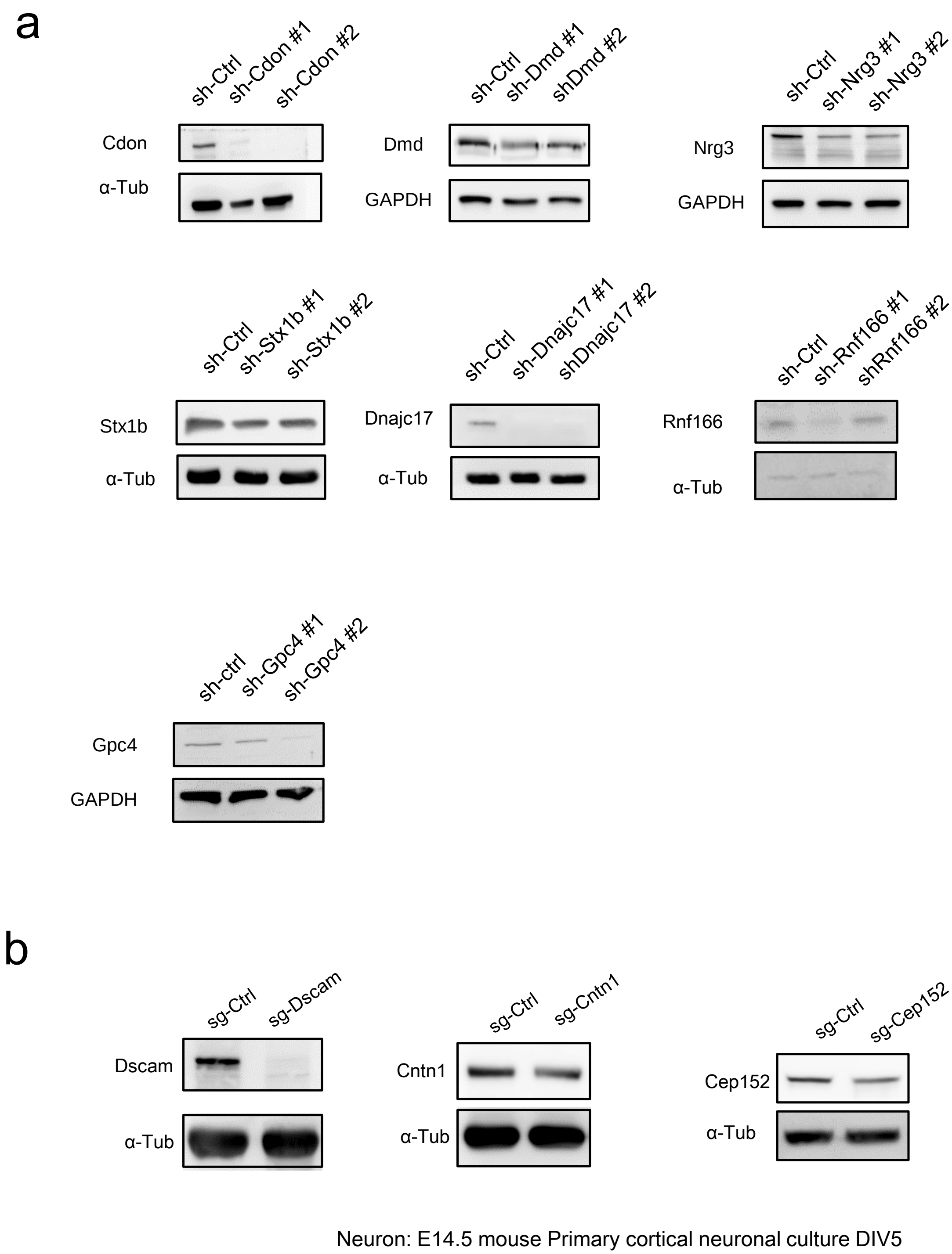

Supplementary Figure 3. Decreases in the protein levels in neurons expressing sgRNA or shRNA against some candidate MCD genes. (a) Primary cultured neurons were infected with lentiviruses encoding shRNA and the expression of the protein was examined 72 hr later by western blotting. (b) Primary cultured neurons were infected with lentiviruses encoding sgRNA and Cas9. The expression of the protein was examined 72 hr later by western blotting. α-tubulin or GAPDH was used as loading control. (c) Western blots shown in **Fig. 5a**. (d), (e) Western blots shown in (a). (f) Western blots shown in (b).

Lu *et al.*, Supplementary Figure 3 (cont.)

c

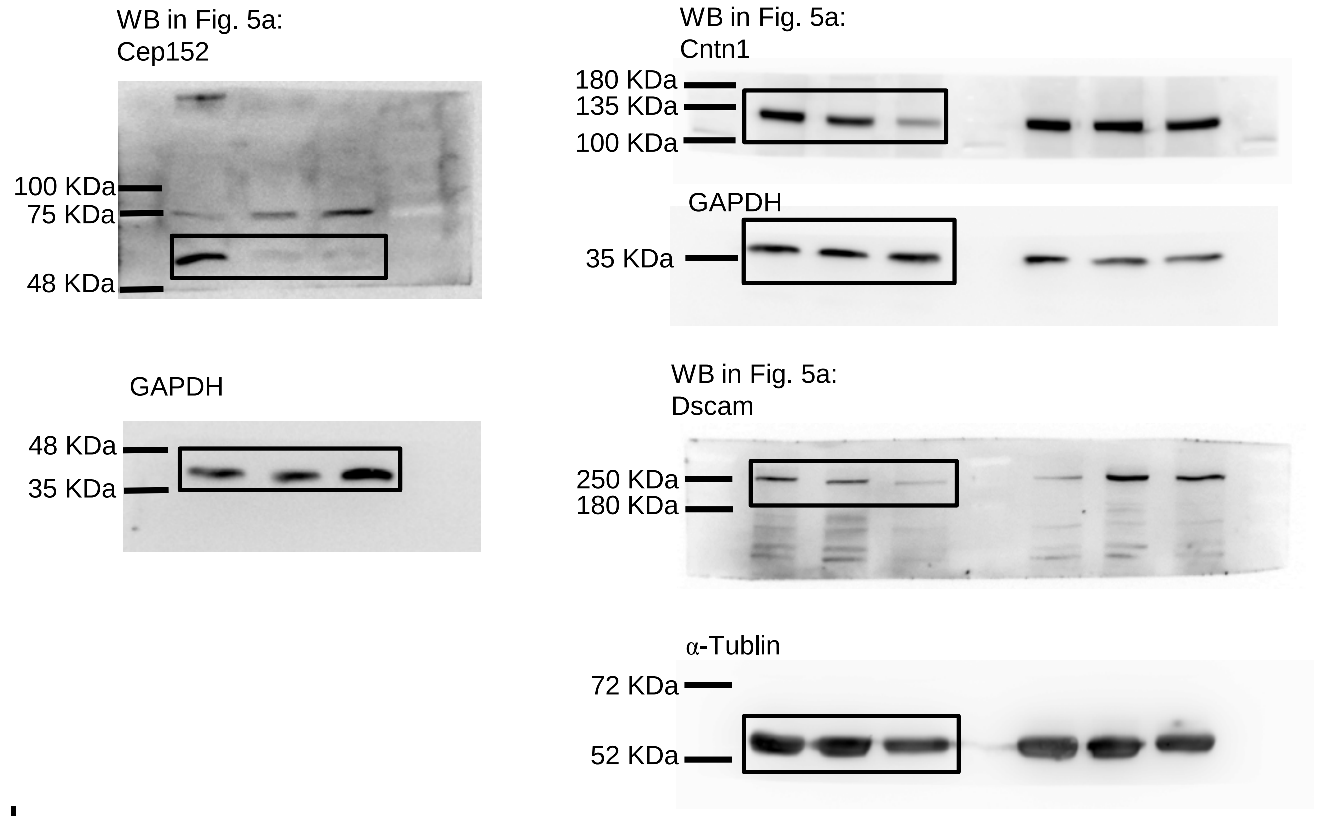

d

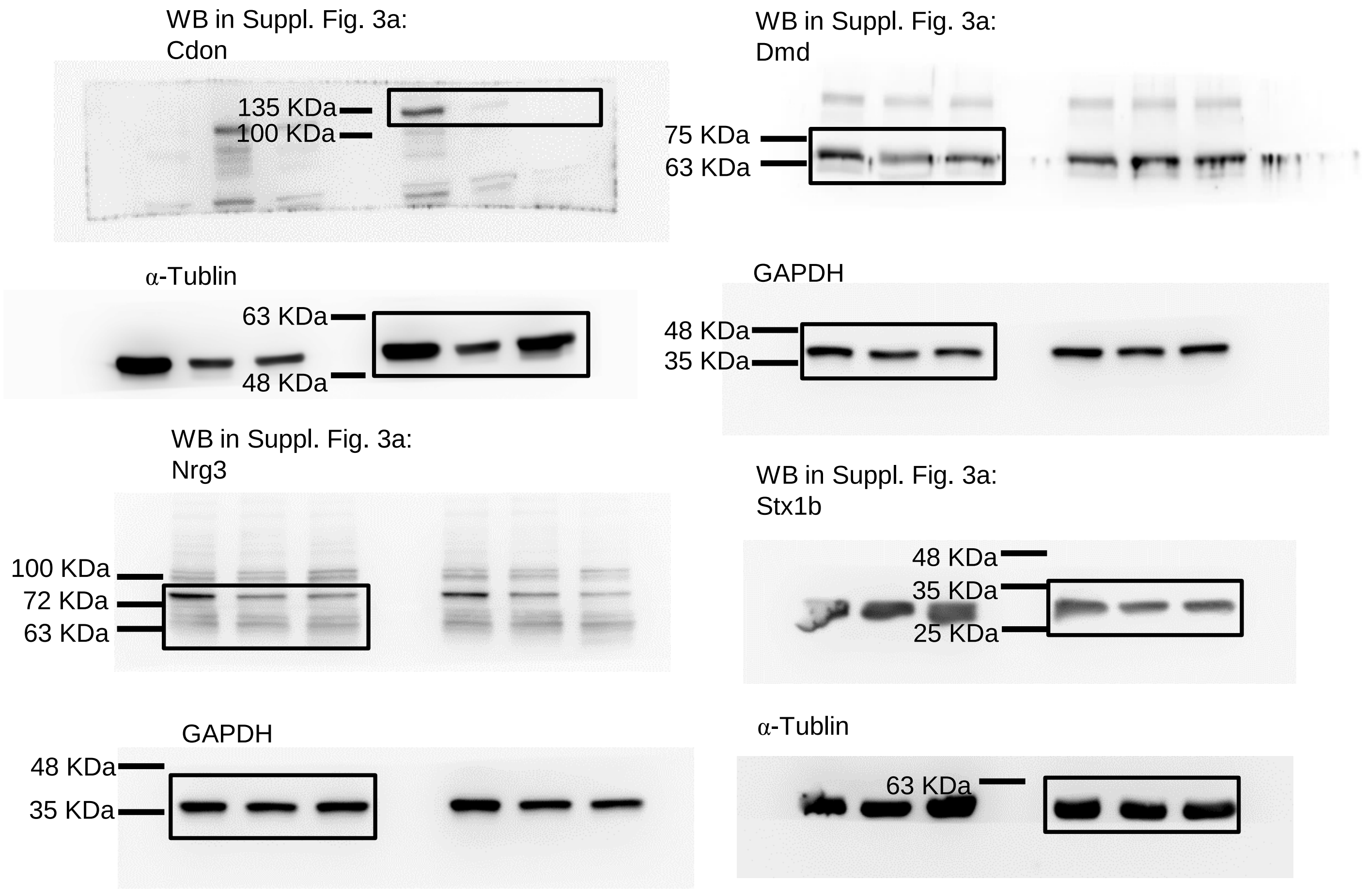

Lu *et al.*, Supplementary Figure 3 (cont.)

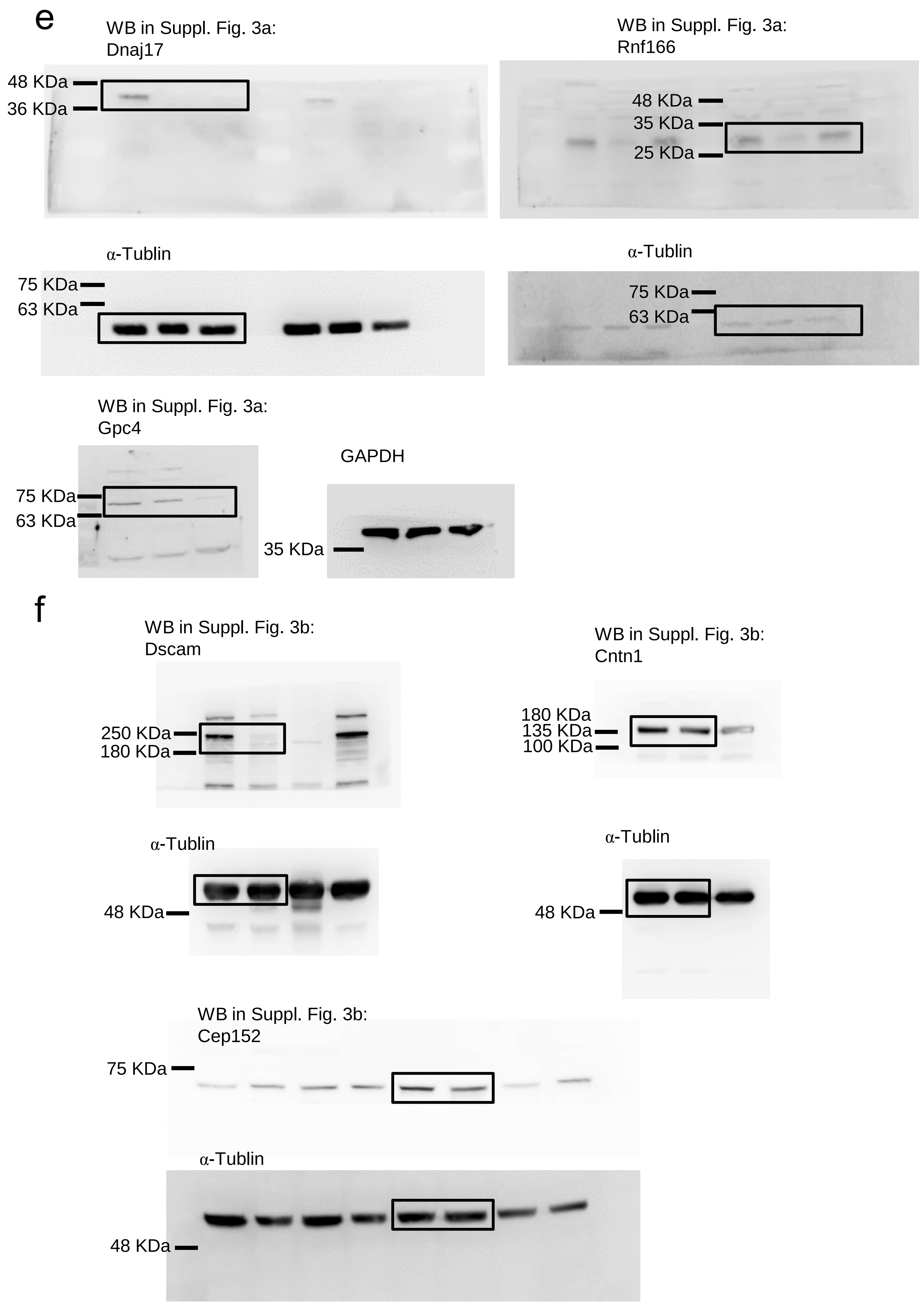

Lu *et al.*, Supplementary Figure 4

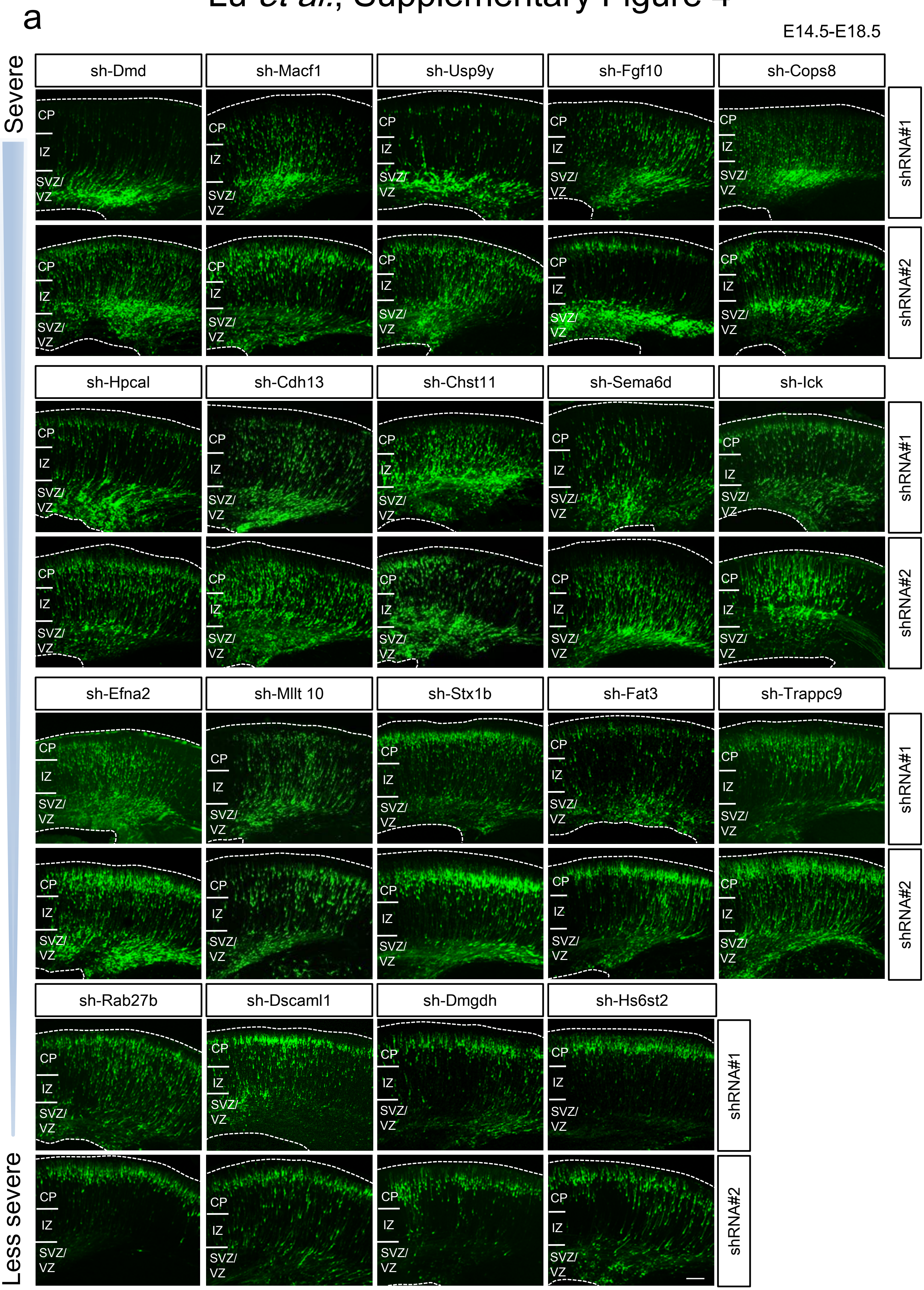

Lu *et al.*, Supplementary Figure 4 (cont.)

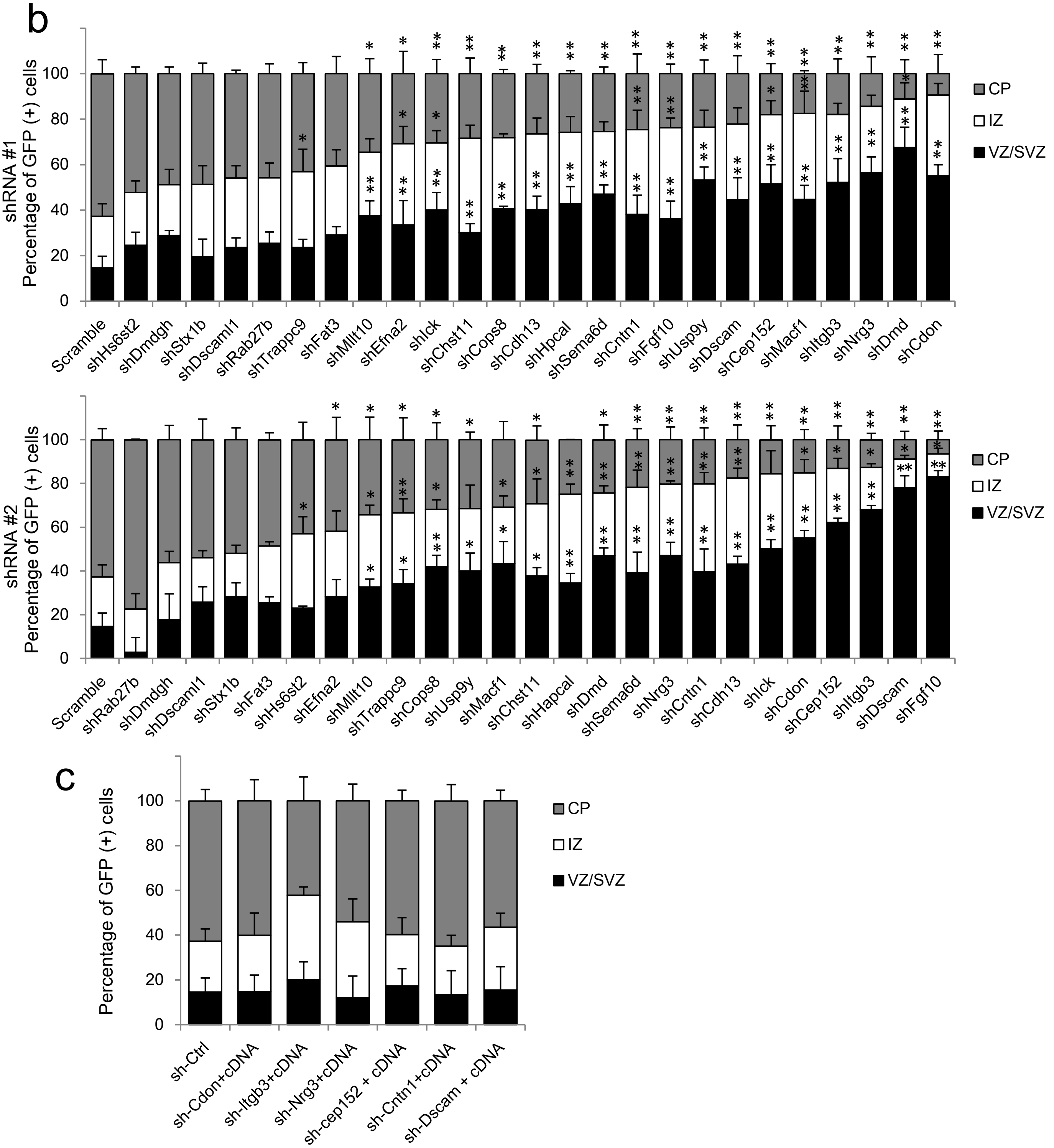

Supplementary Figure 4. The phenotype during brain development after knockdown of additional candidate genes. (a) Cells were electroporated with shRNAs against each candidate genes in the developing neocortex at E14.5. Strong effects on neuron distribution at E18.5 within the cortex were observed after functional knockdown of the genes at the top rows. RNAi against genes at the bottoms rows only produced limited effects. Bar = 100 μm. (b) Bar graphs shows distributions of cells in the brains electroporated with 2 different shRNAs targeting 25 genes from the screening. n = 3 animals for each group. *: $p < 0.05$, **: $p < 0.01$, Student's t test. Error bars = s.d. (c) Rescue of neuronal distribution in brains electroporated with shRNA by expressing their corresponding shRNA-resistant cDNAs. n = 3 animals for each group. Error bars = s.d.

Lu *et al.*, Supplementary Figure 5

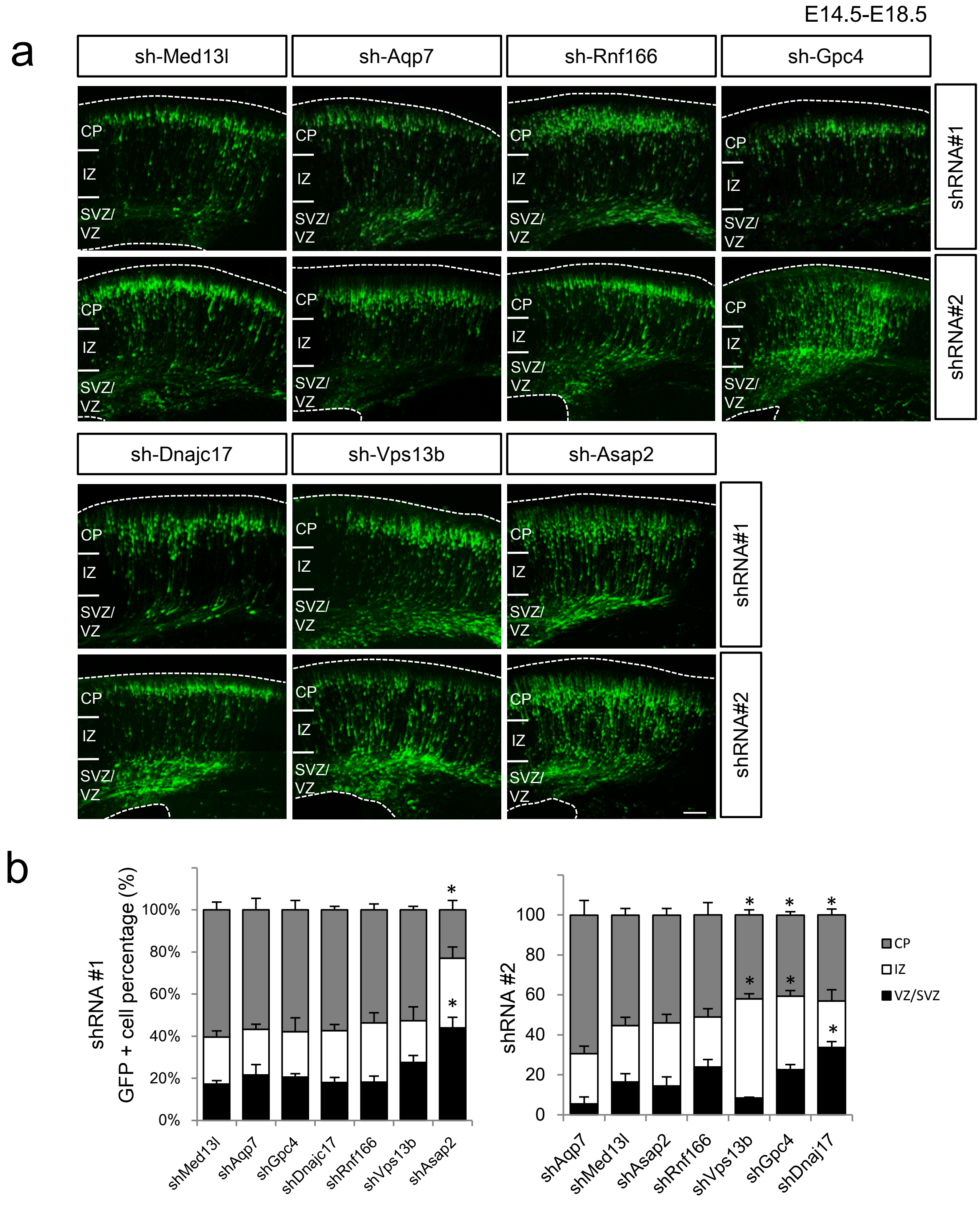

Supplementary Figure 5. The phenotype during brain development at E18.5 after knockdown of additional excluded genes by *in utero* electoporation at E14.5. (a) No or mild effects on neuron distribution within the cortex were observed after functional knockdown for excluded genes. Bar = 100 μm. (b) Distributions of cells in brains electroporated with 2 different shRNAs against each excluded genes. $n = 3$ animals. *: $p < 0.05$, Student's t test. Error bars = s.d.

Lu *et al.*, Supplementary Figure 6

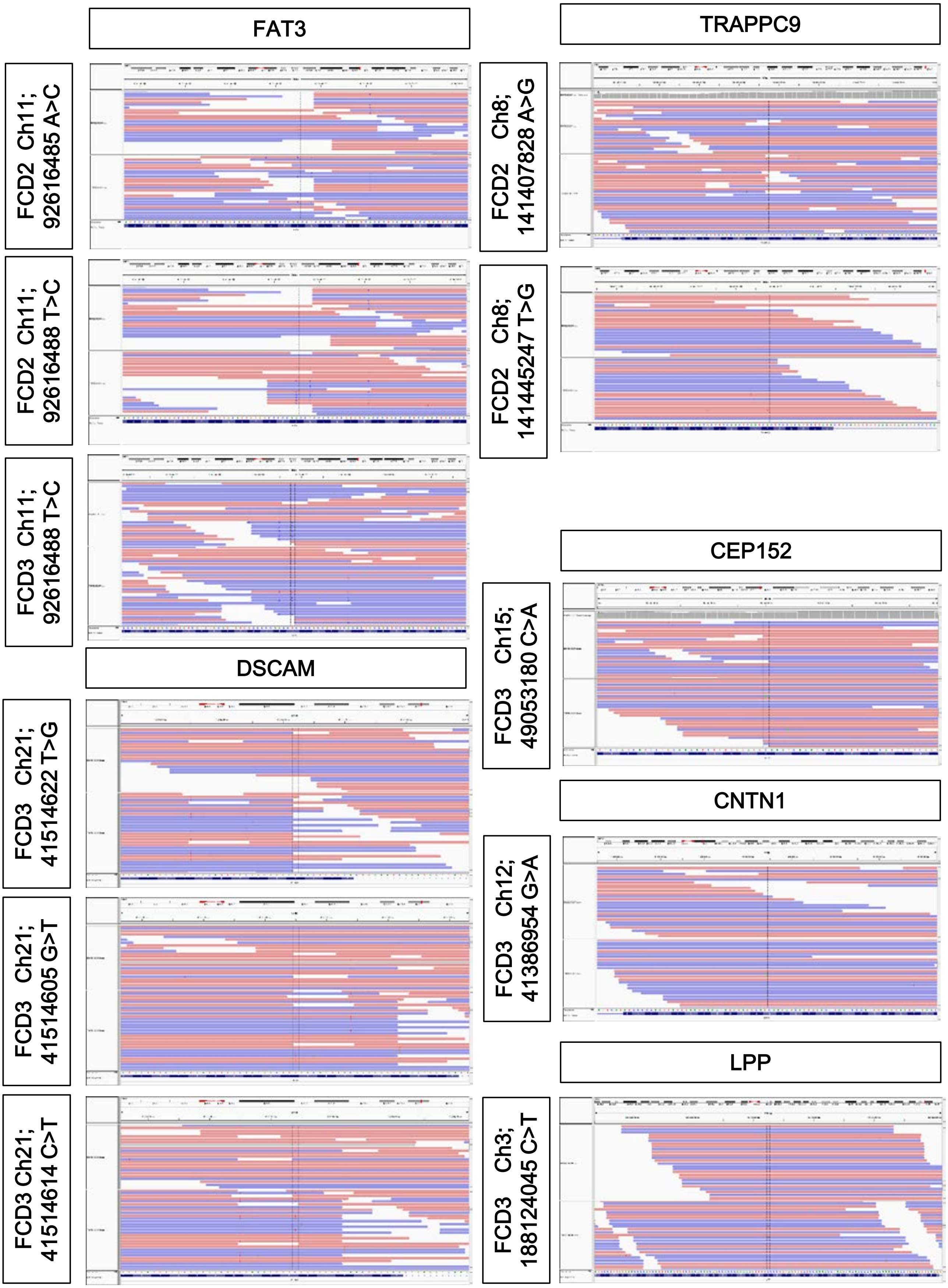

Supplementary Figure 6. Sites of Somatic mutations found in FCD patients. Snapshots of sequencing data from Integrative Genomics Viewer revealing variations found in *FAT3, DSCAM, TRAPC9, CEP152, CNTN1, and LPP*.

Lu *et al.*, Supplementary Figure 7

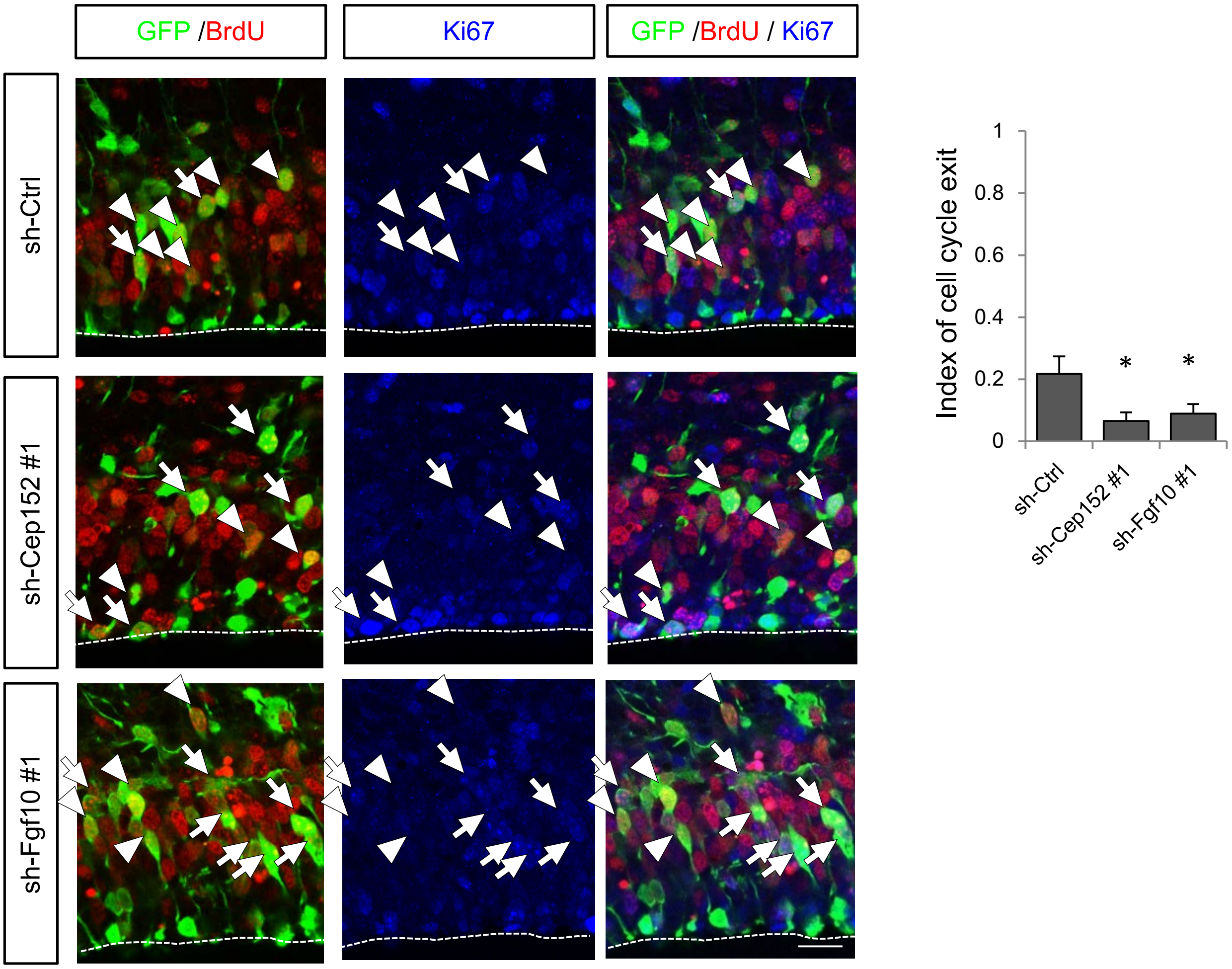

Supplementary Figure 7. Changes in cell cycle exit of neural stem cells in brains electroporated with shRNA targeting Cep152 and Fgf10. Mouse brains were electroporated with Cep152, Fgf10, or control shRNA along with GFP (green) at E13.5 while neural stem cells were labeled with BrdU (red) injection. Cells still undergoing cell cycle were stained with Ki67 (blue, arrows) 24 h later. Arrowheads: cells exiting cell cycle (Ki67-). Bar = 20 µm. Index for cell cycle exit was calculated as the ratio of Ki67- cells among all GFP+/BrdU+ cells (right). n = 3 animals. *: $p < 0.05$, Student's t test. Error bars = s.d.

Supplementary Table 1. Piggybac insertion sites in the mouse genome

Gene name	chr	Location (Start)	Location (End)	Align sequence	Expression		
					Allen brain Atlas	IHC	Ref.
Cops8	1	90612459	90612583	GTGTAATACTGATGGCTCTAACCAA			30
				GCTTGTGGTCGGAGTCCAGTCCTTGACAGAAACTCG			
Teddm2	1	153887165	153887183	GAGGGAGAGGAGGAGGGTG			
Sema6d	2	124610214	124610231	CTCTTCTGTCTGACCGTG	+		
Mllt10	2	18137244	18137281	GGATGGTTTTTTGCTGAG			
Macf1	4	123405745	123405763	GAGAGAGCAATATTTCAAGAATGCATGCG			31
Ppef2	5	92668648	92668760	TATACCCACAAATGGTAGTGATGTAGAGTCGGAAC			
Stx1b	7	127816139	127816462	CTGATTCGGATTTCCTGTTGCAACCTGTAGCTGCTC		+	
Cdh13	8	119289082	119289142	TACGTCCGTCTAGGGGTGAGACCTGGGGCTC	+		
Cdon	9	32700625	32700656	CGGATGTACGCTGCTCTCTTTTCTAGGGTTAAGG	+		
				TCACAGCGTTTGATCGAAGTCTAGATATATGAGTATTT			
Gramd1b	9	40354138	40354219	TCACGGCCAACTTGTTACATGCCGATACTGTTCCATA			
Dscaml1	9	45580292	45580310	TAAAGTGTAAAGCCTGGGGTGCCTAATGAGTGAGCT			32
Fat3	9	15835899	15835972	GAAAGAGAAAGTATGATGCAATCAGAAAGGACACAT	+		
Ick	9	78001578	78001665	TGAACAGAACAAAGGCTCGTGGGCCAAAGCCAGCAG		+	
Efna2	10	80183583	80183658	CTGATGACAGGATGGGCCATGTCTCTTCCTATGAGAG	+		
Chst11	10	83195602	83195630	CTCTCTCTTCTAGGGTTAAAAAAGATTTGCGCTTTAC		+	
Itgb3	11	104648674	104648692	ACACGCAGGCACACAGAA	+		
Hpcal1	12	17785076	17785094	CATTGTATTTGCTCTCTCTTCTAGGGTTAAAAAAGA		+	
Cep152	2	125607960	125607980	ATTGATGATCAGGCCTTGTCTTTTGTTGCACTCTTCC		+	
Dmgdh	13	93686491	93686545	GAGTGGCTCATCAGGTCTGGTGGCAGGATATCTTTT		+	
Fgf10	13	118759172	118759247	CTCTGTATGTGAGAACAGGAACCTATTAGGCCCATG	+		34
LOC667693	13	65780905	65780945	TCCCCGGACACATCCTGTGGTGATTTCCCCAGTTCC			
Nrg3	14	38368933	38368958	GGCTCAGGAGGTGTCTGCGGTGTCTTATTGAGAAC	+		
Ncmap	4	135376763	135376947	TTGGGGTGGTTCATGTGCTGCGCGTACCTCC			
Cntn1	15	92314082	92314108	TAGAATCTTCGTTTGACAAAAACCACATTGTGGCCAA	+		
1700001L05Rik	15	83366351	83366377	CTTTTTCTTTAGTACAGGGAGTGACGA			
Trappc9	15	72637512	72637539	ATGGTTTTTTGCTGAGAAGAGAGGGTGG			33
Slc22a22	15	57449295	57449316	AAATAACAGAAGCCAGAGGGAG			
Dscam	16	96894259	96894287	TTTTGTACGTTGATCTTCTTTCTAGGGTTAAAAAGATTT			32
Lpp	16	24527167	24527185	CAGCTGTGACTGAGACTGTC			
Rab27b	18	70029081	70029102	GGACAGAGGGAGAGGAGGAGGG		+	
Hs6st2	X	51513221	51513241	AGGGAGGGGCTATCTTTCACG			
Dmd	X	83047242	83047260	TGTCCTTTATGTAGAGGAT		+	
Usp9y	Y	1320536	1320560	AACCCCCTGGGCCCCTGGCAATTTTTTAGGAAGAAG		+	

Supplementary Table 2. Piggybac excluded insertion sites in the mouse genome

Gene name	chr	Location (Start)	Location (End)	Align sequence	Expression		
					Allen brain Atlas	IHC	Ref.
Dnajc17	2	119011435	119011390	AGGTGACCTTGCCAGGCTGGCTGGCCCCTGGCTTCTCTTA			
Aqp7	4	40992354	40992376	AGCCACTCACCATCATGACATAGGTA			
NT5C1A	4	122885842	122885865	TCCCCTTTGTGAAGGTGAGGGAGTAGTTCCTGAAACC			
Med13l	5	119043697	119043652	TCCTAAATATAAATTCCAAAATTTACAATTGTTCAAAAAACAT		+	
Rnf166	8	124991159	124991221	CTTCGTCGATGCTGTAGTCCTGGGGAAAAGCAGAGATGCATGA		+	
4933411G06Rik	10	51476018	51476076	TTAACTGATGATGAAAACTAGAACATTCTGAGCTCACATGACA			
Vps13b	15	35401560	35401621	TGAATGTATACCTTTCCCCCTTTTGTAGTACAGTTCCTCTAGTATG		+	
Asap2	15	64310080	64310105	AGACTATGATTCAAGGGTTAAAAAAG			
Gpc4	X	49416051	49416089	TCATCTCTAGTAAAACACAGAACATCAATCAC			

Supplementary Table 3. Top functions from IPA of candidate genes from the genetic screen

Categories	Diseases or functions annotation	p-Value	Molecules	Number of Molecules
Embryonic Development, Organismal Development	development of head	8.93E-05	CDON,CNTN1,DSCAM, EFNA2,FGF10,HS6ST2, TRAPPC9	7
Cellular Assembly and Organization, Cellular Function and Maintenance	organization of cytoskeleton	1.21E-04	CDH13,CNTN1,DSCAM, EFNA2,FGF10,ICK, ITGB3,MACF1	8
Nervous System Development and Function	abnormal morphology of nervous system	1.50E-04	CDON,CNTN1,DSCAM, EFNA2,FGF10,STX1B	6
Cellular Development	differentiation of nervous system	1.72E-04	CDON,CNTN1,DMD, EFNA2,TRAPPC9	5
Nervous System Development and Function, Organ Morphology, Organismal Development	morphology of brain	2.36E-04	CDON,CNTN1,DSCAM, EFNA2,FGF10	5
Cellular Growth and Proliferation, Tissue Development	generation of cells	2.85E-04	CDH13,CHST11,CNTN1, DMD,DSCAM,EFNA2, FGF10,ITGB3,USP9Y	9
Cellular Assembly and Organization, Cellular Function and Maintenance	microtubule dynamics	3.12E-04	CDH13,CNTN1,DSCAM, EFNA2,ICK,ITGB3, MACF1	7
Cellular Assembly and Organization, Nervous System Development and Function	fasciculation of axons	3.17E-04	CNTN1,DSCAM	2

Supplementary Table 4. List of shRNA target sequences used in this study

Gene Name	NM ID	Clone ID	shRNA Number	Target sequence	KD efficiency RNAi core	KD efficiency Neuron WB validation
Cops8	NM_133805	TRCN0000193291	shRNA#1	CTTCAGTTTCAGAAAGCTGTT	85%	
		TRCN0000193308	shRNA#2	CCTGCTATAAAGTCTGCAAAT	70%	
Sema6d	NM_172537	TRCN0000112329	shRNA#1	GCCACCAAACACGGATACAAA		
		TRCN0000112326	shRNA#2	GCAGTCTATAACAGACATAAT		
Mllt10	NM_010804	TRCN0000072134	shRNA#1	CGACTGTTATTTCACAGCCTA		
		TRCN0000072135	shRNA#2	CCCATATGTATGGCAGTAGAT		
Macf1	XM_110503	TRCN0000089573	shRNA#1	CGGTGTGTAAACTCTCTGTTT	72%	
		TRCN0000089577	shRNA#2	CCAGGGTGAATTGATGTTAAA	78%	
Stx1b	NM_024414	TRCN0000110499	shRNA#1	ACTACCAATGAAGAACTAGAA		34%
		TRCN0000110498	shRNA#2	CGACCGAATTGAGTACAACGT		26%
Cdh13	NM_019707	TRCN0000094883	shRNA#1	GCTGCATACACCATCATCAAT	83%	
		TRCN0000094881	shRNA#2	GCAACATCAAACTATCAGGTA	91%	
Cdon	NM_021339	TRCN0000113555	shRNA#1	CCGGAACAACAATAGGTGTTT		40%
		TRCN0000113556	shRNA#2	GCTGTTCTCATCTGCACCATA		65%
Dscaml1	NM_001081270	TRCN0000251164	shRNA#1	ATCCACGACAATGACTATTTC		
		TRCN0000251165	shRNA#2	ACACGCGTGGAGAGATATATA		
Fat3	NM_001017984	TRCN0000097310	shRNA#1	GCAGAGAAACTACTCATTAAA		
		TRCN0000097311	shRNA#2	GCCAAACTTTATGTTCACATT		
Ick	NM_019987	TRCN0000025955	shRNA#1	GCCAAGGCATATACGCTGATT	49%	
		TRCN0000025945	shRNA#2	CCAGTGAAATTGACACAATTT	51%	
Efna2	NM_007909	TRCN0000066288	shRNA#1	CCAACCAATGAGACCCTGTAT		
		TRCN0000066289	shRNA#2	CCTCAAGTTCTCAGAGAAGTT		
Chst11	NM_021439	TRCN0000103331	shRNA#1	CGATGTCAAGTTCGAGGAGTT		
		TRCN0000103332	shRNA#2	CGAAGTCTACAAACTGGACTT		
Itgb3	NM_016780	TRCN0000009618	shRNA#1	CCCTGTTACAATATGAAGAAT	51%	
		TRCN0000009619	shRNA#2	CGCCGTGAATTGTACCTACAA	57%	
Cep152	NM_001081091	TRCN0000251329	shRNA#1	GGAGCTTTGGAGCCGTATAAA		87%
		TRCN0000251328	shRNA#2	TTTAGAGGATCATCGAAATAA		90%
Fgf10	NM_008002	TRCN0000066738	shRNA#1	GCTCTGTAGATCACTAAATAA		
		TRCN0000066739	shRNA#2	CAAATGTATGTGGCATTGAAT		
Cntn1	NM_007727	TRCN0000039018	shRNA#1	CGGCAATCTCTACATCGCAAA		78%
		TRCN0000039016	shRNA#2	GCCTTCAACAATAAAGGAGAT		26%
Trappc9	NM_029640	TRCN0000201938	shRNA#1	CGTGGAGAAGAACGCAACAAA		
		TRCN0000217073	shRNA#2	CAAAGAGGCCATCTCCTATTA		
Dscam	NM_031174	TRCN0000113615	shRNA#1	GCGCAAAGACTACTCTGCTTT		29%
		TRCN0000113616	shRNA#2	CCTGCAATACTCCGAGGATAA		85%
Dmd	NM_007868	TRCN0000077146	shRNA#1	CGGAAGTAAATCTGGATAGTT	48%	27%
		TRCN0000077147	shRNA#2	GCGGCAAATTGAGAGCAATTT	64%	34%
Nrg3	NM_008734	TRCN0000065403	shRNA#1	GCTGTCAATTTCATGTATCAT		48%
		TRCN0000065404	shRNA#2	CCAGCATATCAACAACTTGAA		62%
Dmgdh	NM_028772	TRCN0000041920	shRNA#1	CCCGGGATAAACTTGAAGAAA		
		TRCN0000041919	shRNA#2	GCCACGGAACAGTATATTATT		
Usp9y	NM_148943	TRCN0000030854	shRNA#1	GCTCTTACTTTACAGGATCTT	46%	
		TRCN0000030855	shRNA#2	TAGTGATTTACACGATGATAT	47%	
Hs6st2	NM_015819	TRCN0000103147	shRNA#1	CGCCGTTTACCCAGTATAATA	70%	
		TRCN0000103148	shRNA#2	GCTAAAGACCTCTTTCTGCAA	70%	
Hpcal	NM_016677	TRCN0000327206	shRNA#1	GACTGTGGACGAGTTCAAGAA	90%	
		TRCN0000306646	shRNA#2	TTTAGCATGTACGACCTGGAC	60%	
Rab27b	NM_030554	TRCN0000100426	shRNA#1	CGGGAAGACAACATTTCTCTA	87%	
		TRCN0000100427	shRNA#2	GCATACCATACTTCGAAACAA	88%	

Supplementary Table 4. List of shRNA target sequences used in this study (Cont.)

Gene Name	NM ID	Clone ID	shRNA Number	Target sequence	KD efficiency	
					RNAi core	Neuron WB validation
Rnf166	NM_001033142	TRCN0000238847	shRNA#1	TGGTGAAGCACTGCGTGGAAA	82%	87%
		TRCN0000238846	shRNA#2	TGATCCCAACCGTGTGGTATG	84%	64%
Vps13b	NM_177151	TRCN0000256669	shRNA#1	ATCGTGCATTCATGGATATTT	48%	
		TRCN0000256666	shRNA#2	ACCTATGTTTATCCGTATAAT	49%	
Med13l	NM_172424	TRCN0000111895	shRNA#1	CATGCTCTAACCGGGAAGGGA	74%	
		TRCN0000111897	shRNA#2	CCCAGTCCAATCCAGCTTTAT	79%	
Asap2	NM_001004364	TRCN0000103986	shRNA#1	GCCTCCATTGAGATAGCCAAT	63%	
		TRCN0000103988	shRNA#2	GCCCTTTGATAAAGCTTGGAA	63%	
Dnajc17	NM_139139	TRCN0000120972	shRNA#1	GCATATGACAAGGTTAGGAAA	86%	99%
		TRCN0000120976	shRNA#2	AGGAAGAAAGTGAAACTTGAT	85%	80%
Aqp7	NM_007473	TRCN0000102162	shRNA#1	CCAGAAGGTAACTGCATCATA		
		TRCN0000102163	shRNA#2	CCGGATATGCAATCAACCCAT		
Gpc4	NM_008150	TRCN0000109465	shRNA#1	GCCACTGGTTTAAGCAATGTT	90%	20%
		TRCN0000109466	shRNA#2	GCATCCCGTTACAAGAAGTTT	88%	44%
Scramble Sequence (TRC1)		ASN0000000004		CGGTGTGTAAACTCTCTGTTT		
Scramble Sequence (TRC2)		ASN0000000003		CCTAAGGTTAAGTCGCCCTCG		

Neuron: E14.5 mouse Primary cortical neuronal culture DIV5

Supplementary Table 5. Summary of WES coverage and depth

Sample	Total length of targets with coverage below 30	Average coverage	Coverage of target regions (%)					
			5X	10X	20X	40X	80X	100X
**FCD1-Blood**	25531044	152.4	88.56	87.01	84.59	79.97	68.57	61.9
**FCD2-Blood**	13933018	53.59	95.54	92.43	83.70	59.94	20.78	10.70
**FCD3-Blood**	16775387	53.19	95.35	91.42	80.15	53.70	20.33	12.45
**FCD4-Blood**	14158433	52.6	95.43	92.26	83.42	59.24	19.82	9.93
**FCD5-Blood**	25006250	151.0	89.88	88.49	86.17	81.37	69.18	62.1
**FCD6-Blood**	22179236	186.7	89.92	88.6	86.61	83.00	74.53	69.39
**FCD1-Tissue**	28192020	122.9	87.96	86.14	83.21	77.19	61.86	53.28
**FCD2-Tissue**	5969121	109.63	96.48	95.18	92.04	83.63	60.96	49.20
**FCD3-Tissue**	6334256	106.69	96.82	95.21	91.73	82.39	58.33	46.47
**FCD4-Tissue**	6072611	110.95	96.54	95.13	91.87	83.41	61.06	49.52
**FCD5-Tissue**	31049698	109.7	87.8	85.84	82.5	75.42	57.68	48.09
**FCD6-Tissue**	29522516	114.0	87.18	85.25	82.12	75.68	59.05	49.96

Supplementary Table 6. Summary of number of genetic variants in each filtering step

Patients	FCD1	FCD2	FCD3	FCD4	FCD5	FCD6	Total Numbers
Variants (Mutect2/Virmid)	10845 /85358	5141 /35486	9469 /54203	6859 /44483	10234 /76877	10115 /69459	
Union of Variants	96203	40627	63672	51342	87111	79573	347808
Functional filtering	1882	3335	3882	3749	2257	1739	14133
Qualitative filtering (DP≥20,AC≥4)	380	1197	1718	1313	394	368	3605
Gene Mapping	238	1014	1439	1094	235	214	2531
Candidate Gene Crossing	0	2	2	2	0	0	6

Supplementary Table 7. List of sgRNA target sequences used in this study

Gene Name	NM ID	species	Target sequence	Neuron KD efficiency(%)	
				mRNA	Protein
Cep152	NC_000068.7	mouse	GTCCAATTAGAAATAGCAAT<br>GGAAGAGTGCAACAAAAGAC	78	41.5
Cntn1	NC_000081.6	mouse	GTGTGCATCCTAGAATGCAC<br>GTCATTAGCCAGTGCATTCT	27	21.5
Dscam	NC_000082.6	mouse	GTCAACGCACAAGACAAGAG<br>GACGCACAAGACAAGAGAGG	35	78

Neuron: E14.5 mouse Primary cortical neuronal culture DIV5
