# Supplementary material for: Identification of genes associated with cortical malformation using a transposon-mediated somatic mutagenesis screen in mice
Source: Nat Commun. 2018 Jun 27;9:2498. doi: 10.1038/s41467-018-04880-8 (PMC6021418; doi:10.1038/s41467-018-04880-8)
Supplement: Supplementary file 2 — Description of Additional Supplementary Files [file 41467_2018_4880_MOESM2_ESM.pdf]

Description of Additional Supplementary Files

File Name: Supplementary Data 1
Description: PB insertion sites in normotopic brain tissue.

File Name: Supplementary Data 2
Description: IPA analysis of candidate genes: Disease and biological functions.

File Name: Supplementary Data 3
Description: Variants detected by Mutect and Virmid.

File Name: Supplementary Data 4
Description: Mutations of candidate genes found in FCD patients.
